# The infection–microbiome–immunity axis in bladder cancer: mechanistic insights and therapeutic perspectives

**DOI:** 10.3389/fimmu.2025.1716230

**Published:** 2026-01-12

**Authors:** Shen Pan, Wanlin Cui, Jiaman Lin, Zhujun Wang, Zhenhua Li, Bitian Liu

**Affiliations:** 1 Department of Nuclear Medicine, Shengjing Hospital of China Medical University, Shenyang, China; 2 Department of Pediatrics, The First Affiliated Hospital of China Medical University, Shenyang, Liaoning, China; 3 Department of Urology, Shengjing Hospital of China Medical University, Shenyang, China; 4 Department of Microbiology and Immunology, Keio University School of Medicine, Tokyo, Japan

**Keywords:** bladder cancer, immunosenescence, inflammation, microbiome, urinary tract infections

## Abstract

Bladder cancer (BC) represents a paradigm of infection-associated malignancy in which microbial dysbiosis, immune aging, and tumor microenvironmental remodeling converge to shape disease progression. Increasing evidence highlights the dual role of the urinary and gut microbiota in modulating bladder carcinogenesis through infection-driven inflammation and immune dysfunction. Chronic exposure to uropathogens and microbial imbalance disrupts epithelial integrity, promotes extracellular matrix degradation, and reprograms local immune signaling, collectively fostering a tumor-permissive niche. Concurrently, immunosenescence exacerbates microbial persistence and impairs antitumor immunity, reinforcing a pathogenic feedback loop between infection and immune decline. This review integrates current insights from microbiome research, tumor immunology, and microbial pathogenesis to delineate the mechanistic continuum linking infection, dysbiosis, and immune remodeling in BC. Finally, we discuss emerging microbiome-targeted and immunomodulatory strategies aimed at restoring microbial–immune equilibrium and improving therapeutic efficacy. Together, these perspectives provide a refined conceptual framework for understanding infection-driven oncogenesis and guiding precision interventions in BC.

## 1 Introduction

Bladder cancer (BC) is the second most common genitourinary malignancy globally, with around 550,000 new cases and 200,000 deaths each year (1). Despite significant advances in surgical and systemic therapies, BC continues to exhibit high recurrence rates and poor survival, particularly in advanced or muscle-invasive disease. For advanced BC, the prognosis remains poor, with a 5-year survival rate below 40% (2). This persistent clinical burden underscores the need to better understand non-genetic and microenvironmental factors that shape tumor initiation and progression. In addition to microbial and immune factors, key environmental risk factors for BC include tobacco smoking, which accounts for approximately 50% of cases in developed countries, and occupational exposure to aromatic amines and other toxic chemicals (e.g., in dye, rubber, and leather industries), which contribute to 5-10% of cases. These exposures can induce DNA damage and chronic inflammation, potentially interacting with the urinary microbiome by promoting dysbiosis—such as reduced microbial diversity and enrichment of pro-inflammatory taxa like Proteobacteria—which may exacerbate infection-driven carcinogenesis (3, 4). Exploring these links could reveal how smoking-induced alterations in gut and urinary microbiota amplify oxidative stress and immune dysregulation in the bladder. In recent years, the human microbiome has emerged as a critical determinant of oncogenesis, influencing cancer risk, immune modulation, and therapeutic outcomes across multiple organ systems. The discovery of a resident microbiota within the urinary tract has challenged the long-held assumption of its sterility, paving the way for new research exploring the role of host-microbe interactions in bladder carcinogenesis.

**Figure 1:**
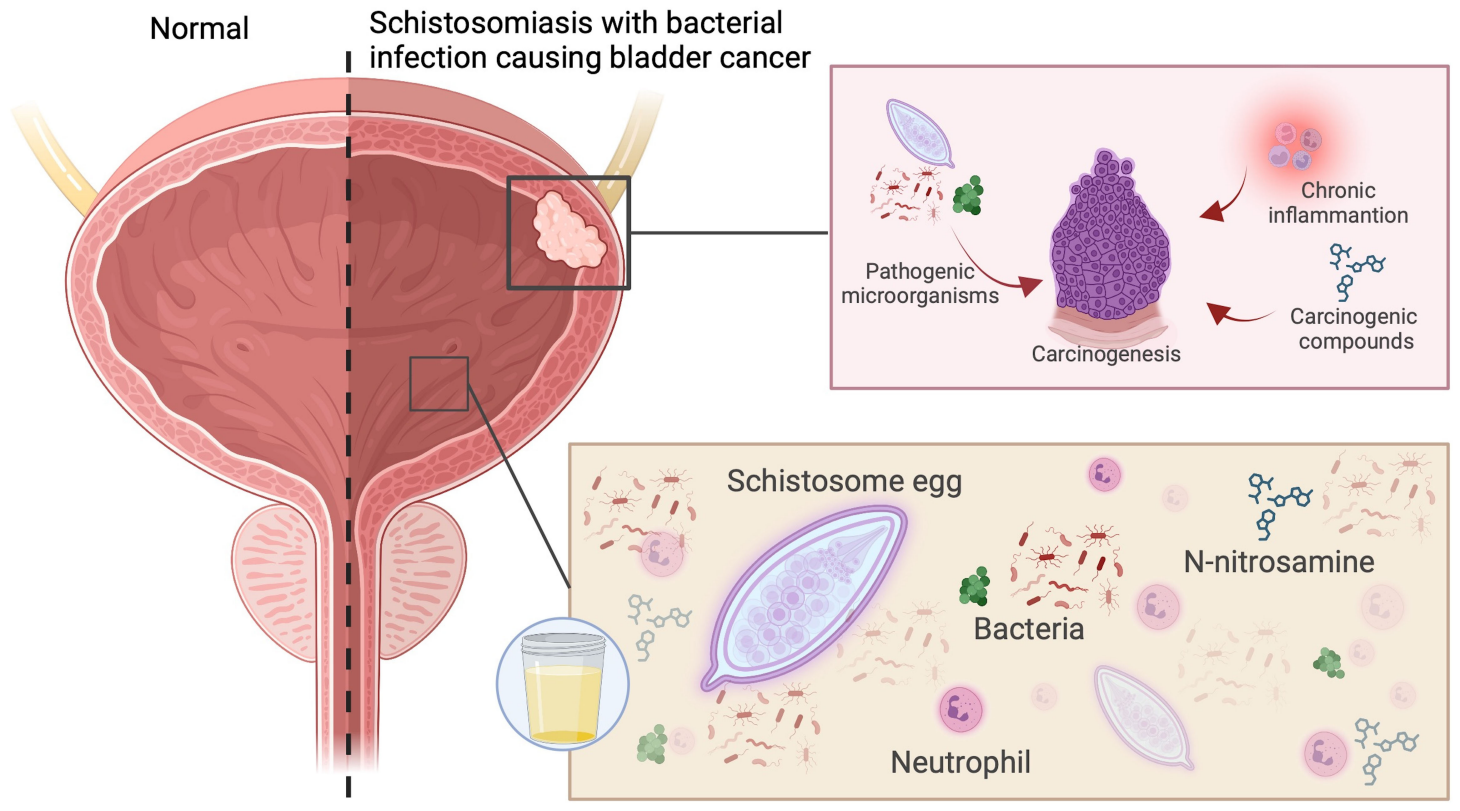
Schistosoma infection is often accompanied by microbial infections, which can trigger chronic inflammation and the accumulation of harmful substances, collectively contributing to the development of bladder cancer.

Historically, the role of infection in BC was recognized in regions endemic for Schistosoma haematobium, where chronic parasitic inflammation predisposes to squamous cell carcinoma of the bladder (5–7). Beyond schistosomiasis, growing evidence indicates that bacterial infections—particularly recurrent urinary tract infections (UTIs)—contribute to urothelial carcinogenesis through persistent inflammation, oxidative stress, and epithelial injury. Uropathogenic Escherichia coli (UPEC), responsible for over 70% of UTIs, can invade bladder epithelial cells, form intracellular bacterial communities, and establish chronic reservoirs that are resistant to host clearance. These persistent infections elicit prolonged inflammatory signaling and reactive oxygen species (ROS) production, both of which promote DNA damage and mutagenesis (8–10). Moreover, interactions between schistosomes and cohabiting bacteria—such as *Fusobacterium*, *Sphingobacterium*, and *Enterococcus*—can enhance carcinogenicity through the generation of N-nitrosamines and estrogen-like DNA-reactive metabolites (11–13) (**Figure 1**). Epidemiologic studies further suggest that recurrent UTIs, pyuria, and chronic cystitis correlate with increased risk of BC recurrence and progression, reinforcing the clinical relevance of infection-mediated pathways.

An additional layer of complexity arises from aging-related immune decline, or immunosenescence. Bladder cancer predominantly affects older adults, with a median age at diagnosis of approximately 73 years (14). Aging is accompanied by diminished immune surveillance, reduced antigen presentation, and impaired cytotoxic T-cell responses, which collectively reduce the host’s ability to eliminate pathogens and emerging tumor cells (2, 15, 16). Immunosenescence also facilitates microbial persistence, establishing a vicious cycle in which chronic infection perpetuates inflammation, and inflammation accelerates immune deterioration (17–21). This interplay between microbial dysbiosis and immune aging represents a critical yet underexplored determinant of BC pathogenesis. Understanding this dynamic crosstalk may reveal why elderly individuals are more susceptible to both infection and cancer, and why immune-based therapies exhibit variable efficacy in this population.

The duality of microbes as both carcinogenic agents and therapeutic tools further highlights the complexity of host–microbe interactions in BC. The attenuated strain Mycobacterium bovis Bacillus Calmette–Guérin (BCG) remains the cornerstone of intravesical therapy for non–muscle-invasive bladder cancer (NMIBC), harnessing microbial activation of innate and adaptive immunity to suppress tumor recurrence (22). This paradox—where microbes can either promote or suppress tumorigenesis—underscores the necessity of disentangling the mechanistic nuances governing microbial behavior within the bladder milieu. Clarifying the context-dependent effects of microbial exposure is essential to advancing precision interventions that exploit beneficial microbial functions while mitigating pathogenic consequences (23).

In this context, the present review aims to synthesize current knowledge on the microbiome–infection axis in bladder cancer and to propose a unifying conceptual framework linking microbial dysbiosis, chronic inflammation, and immunosenescence to tumor initiation and progression. We first examine evidence from clinical and experimental studies delineating how infection-driven inflammatory signaling contributes to urothelial carcinogenesis. We then explore the composition and ecological dynamics of urinary and gut microbiota in BC, identifying microbial signatures associated with disease progression and therapeutic response. Subsequently, we integrate mechanistic insights into how microbes and immune aging remodel the tumor microenvironment (TME) through epithelial barrier disruption, immune reprogramming, and extracellular matrix modification. Finally, we discuss therapeutic implications, emphasizing microbiome-targeted and immunomodulatory strategies that hold promise for restoring microbial–immune homeostasis and improving clinical outcomes.

By bridging microbiology, immunology, and cancer biology, this review positions the microbiome not as a peripheral feature but as a central determinant of bladder cancer pathogenesis. Understanding the intricate relationship between infection, microbial ecology, and host immunity will be essential for developing next-generation diagnostic tools and personalized therapeutic strategies that move beyond the tumor-centric paradigm toward a holistic model of host–microbe–tumor interaction.

## 2 Infection-driven microbiome dysregulation in BC

Chronic and recurrent infections are among the most significant environmental pressures shaping the bladder’s microbial ecosystem and influencing tumor initiation. The urothelial surface, once thought to be sterile, is now recognized as a dynamic microbial niche whose composition is constantly modulated by host immunity, urinary flow, and exposure to pathogens. When these regulatory mechanisms fail—particularly under conditions of persistent infection or age-related immune decline—the resulting microbial imbalance, or dysbiosis, can initiate a cascade of inflammatory and mutagenic processes that contribute to bladder carcinogenesis.

### 2.1 Pathogenic infections and carcinogenic inflammation

Chronic infection by *E. coli*, the primary pathogen responsible for 70% of UTIs, has been implicated as a potential carcinogenic factor. Histopathological studies show that persistent *E. coli* infection alone induces epithelial dysplasia in the mucosal lining and promotes inflammatory cell infiltration into the lamina propria. When combined with nitrosamine precursors, chronic infection significantly increases the incidence of bladder lesions, surpassing the effects of nitrosamine precursors alone. This synergy highlights the critical role of infection-driven inflammation in bladder carcinogenesis (17). Kawai et al. further demonstrated that E. coli-derived lipopolysaccharide (LPS) significantly enhances N-methyl-N-nitrosourea (MNU)-induced bladder carcinogenesis, with inflammation and oxidative stress, driven by reactive oxygen species (ROS), playing key roles in this process (24). However, these findings, largely based on animal models, may not fully capture the complexities of human BC. Additionally, genetic and environmental variations limit the direct applicability of these results to clinical settings. Therefore, future research should focus on validating these mechanisms in human populations while accounting for these influencing factors.

### 2.2 UTIs and prognosis of BC

The connection between inflammation and bladder cancer (BC) progression is well-established. Sazuka et al. found that preoperative pyuria is closely associated with intravesical recurrence after transurethral resection of bladder tumor (TURBT), suggesting its role in predicting recurrence risk (19). Similarly, Jing et al. highlighted the importance of tumor-neutrophil interactions within the TME as a key driver of BC progression, emphasizing the critical role of inflammation in this process (25). Abd-El-Raouf et al. demonstrated that *E. coli* infection accelerates BC progression by inducing epithelial-mesenchymal transition (EMT), stem cell-like behaviors, and metabolic reprogramming (26). Nesi et al., in their systematic review, identified chronic inflammation as a central mechanism in BC pathophysiology (27), while Russell et al. further supported the inflammatory hypothesis by showing that epigenetic reprogramming induced by uropathogenic *E. coli* influences BC outcomes (28). Although the role of inflammation in BC progression is widely recognized, there remains some disagreement on its exact contribution. While some studies focus on the direct effects of inflammation on tumorigenesis, others suggest a more complex interplay involving microbial dysbiosis and immune suppression. Furthermore, most studies rely on mechanistic or single-center data, which may oversimplify the multifactorial nature of BC progression. To address these discrepancies, future research should adopt longitudinal studies with integrated approaches that assess inflammation, microbial composition, and tumor dynamics across diverse populations.

### 2.3 Prognostic biomarkers in infection-associated BC

Clinical studies have identified pyuria and recurrent UTIs as negative prognostic factors in BC patients. Vermeulen et al. reported that recurrent UTIs significantly increase the risk of developing BC (29). Azuma et al. identified pyuria as a poor prognostic indicator in patients with NMIBC and found it correlated with lower survival rates (21). Similarly, Singh et al. showed that preoperative pyuria and an elevated neutrophil-to-lymphocyte ratio (NLR) independently predict poor outcomes (18). Satake et al. also highlighted the prognostic value of preoperative pyuria in NMIBC patients (20). Although these studies emphasize the relevance of pyuria and recurrent UTIs as prognostic markers, inconsistencies remain in sample sizes and study designs. Many rely on single-center populations or small cohorts, which may introduce sampling bias and limit the generalizability of the results. To improve the accuracy of prognostic assessments, future studies should adopt multicenter designs with larger and more diverse populations to better define the value of these biomarkers across different clinical and demographic contexts.

## 3 Microbiome composition and its clinical correlates

The recognition that the bladder harbors a distinct microbial ecosystem has fundamentally transformed our understanding of urinary tract biology and its contribution to disease. Advances in metagenomic sequencing and culture-independent techniques have revealed that both the urinary and gut microbiota undergo profound compositional shifts during BC development. These alterations—manifesting as reduced microbial diversity, loss of beneficial commensals, and enrichment of opportunistic pathogens—reflect a disrupted ecological equilibrium, or dysbiosis, that can influence immune homeostasis, inflammatory signaling, and tumor behavior. Importantly, the specific microbial signatures observed in urine, tissue, and stool samples from BC patients provide valuable insights into the pathophysiology of the disease and its potential diagnostic and prognostic biomarkers (12, 30–35) (**Table 1**).

**Table 1:** Studies of urinary microbiome in bladder cancer.

Study	Year	BCa	Non-BCa	Sample	Alpha diversity	Beta diversity	Increase in abundance/phyla	Increase in abundance/genera	Study purpose
Bučević Popović et al. (36)	2018	12	11	Mid-stream urine	NS	NS	Actinobacteria, Proteobacteria (BC); Firmicutes, Bacteroidetes (controls)	Fusobacterium, Streptococcus, Peptoniphilus (BC); Corynebacterium Prevotella (controls)	BC vs. healthy controls
Wu et al. (37)	2018	31 (26 NMIBC and 5 MIBC)	18	Mid-stream urine	Different	Different	Proteobacteria, Firmicutes, Actinobacteria, Bacteroidetes (BC)	Acinetobacter, Anaerococcus, Rubrobacter, Sphingobacterium (BC)	BC vs. healthy controls
Bi et al. (38)	2019	29	26	Mid-stream urine	Different	Different	Tenericutes, Proteobacteria (BC)	Actinomyces (BC)	BC vs. healthy controls
Liu et al. (41)	2019	22 (17 MIBC and 5 NMIBC)	12	Tissue	Different (Shannon index)	Different	Proteobacteria, Actinobacteria (BC); Firmicutes, Bacteroidetes (non-tumor tissue)	Escherichia-Shigella, Acinetobacter, Ralstonia, Cupriavidus, Pelomonas, Sphingomonas (BC); Lactobacillus, Prevotella_9, Ruminococcaceae (non-tumor tissue)	BC vs. adjacent non-cancerous
Chipollini et al. (35)	2020	27 (15 MIBC and 12 NMIBC)	10	Mid-stream urine	Different	NS	NR	Bacteroides and Faecalbacterium (BC)	BC vs. healthy controls
Zeng et al. (39)	2020	62 males (51 NMIBC and 11 MIBC)	19	Mid-stream urine	Different	Different	Proteobacteria, Actinobacteria (BC)	Acinetobacter, Anaerococcus, Sphingobacterium (BC) Micrococcus, Brachybacterium (recurrence)	BC vs. controls; recurrence analysis
Mansour et al. (42)	2020	10 urine, 14 tumor tissues		Mid-stream urine Tissue	NS	NS	Firmicutes, Proteobacteria (BC tissues); Firmicutes, Actinobacteria, Cyanobacteria, Bacteroidetes (urine)	Akkermansia, Bacteroides, Clostridium sensu stricto, Enterobacter, Klebsiella (BC tissues); Lactobacillus, Corynebacterium, Streptococcus, Actinomyces (urine)	BC tissues vs. urine samples
Pederzoli et al. (44)	2020	49	59	Mid-stream urine Tissue	NS	Different (BC vs. Controls in Female urine)	Klebsiella (female BC urine); Burkholderia (BC tissue)	Not provided	Sex-based microbiota differences
Hussein et al. (40)	2021	43 (29 NMIBC and 14 MIBC)	10	Mid-stream urine (healthy), Cath/cystoscopy (cancer patients)	NS	Different (BC vs. controls)	Proteobacteria (NMIBC); Firmicutes, Proteobacteria (MIBC); Firmicutes, Proteobacteria (BCG responders)	Cupriavidus (NMIBC); Hemophilus, Veillonella (MIBC); Serratia, Brochothrix, Negativicoccus, Escherichia-Shigella, Pseudomonas (BCG responders)	NMIBC vs. MIBC; BCG post-therapy recurrence analysis
Parra-Grande et al. (43)	2021	32	26	Tissue	Different	NR	Proteobacteria, Fusobacteria (BC) Actinobacteria (non-tumor tissue)	Fusobacterium, Barnesiella, Escherichia-Shigella (BC) Staphylococcus, Lactobacillus, Actinomyces (non-tumor tissue)	BC vs. adjacent non-cancerous
Oresta et al. (46)	2021	51	10	Mid-stream urine and catharized urine	Different (MIBC vs. NMIBC)	Different (MIBC vs. NMIBC)	Proteobacteria, Firmicutes (NMIBC)	Veillonella, Corynebacterium, Ruminococcus (NMIBC)	MIBC vs. NMIBC
Qiu et al. (51)	2022	40 (12 recurrent, 28 non-recurrent NMIBC)		Mid-stream urine	Different (Higher alpha diversity in recurrent)	Different	Proteobacteria, Actinobacteria, Firmicutes (recurrence)	Pseudomonas, Acinetobacter, Staphylococcus, Corynebacterium (recurrence)	Recurrence analysis
Sun et al. (47)	2023	22 (15 MIBC, 7 NMIBC)		Tissue	Different	Different	Proteobacteria (dominant in MIBC)	Ralstonia (dominant in MIBC)	MIBC vs. NMIBC
Hussein et al. (50)	2023	68 (26 non-recurrent and 42 recurrent)		Urine	NS	Different	NR	Escherichia-Shigella, Helococcus (BCG responders); Veillonella, Bifidobacterium (BCG non-responders)	BCG post-therapy recurrence analysis
Bilski et al. (48)	2024	41 (21 males and 20 females) urine 10 tissues	10 tissues	Mid-stream urine Tissue	Different	Different	Proteobacteria (male urine), Firmicutes (female urine); Proteobacteria (BC tissue), Firmicutes (non-tumor tissue)	Escherichia (male urine), Streptococcus (female urine); Fusobacterium, Prevotella (BC tissue), Lactobacillus (non-tumor tissue)	Sex-based microbiota differences (urine) BC vs. adjacent non-cancerous (tissue)
Yao et al. (45)	2024	22	22	Tissue	Different (except for BCG therapy)	Different (except for BCG therapy)	Proteobacteria (BC); Actinobacteria (non-tumor tissue)	Pseudomonas, Porphyrobacter, Acinetobacter (BC); Staphylococcus, Lactobacillus (non-tumor tissue); Erythrobacter, Corynebacterium, Streptomyces, Mycolicibacterium (BCG durable responders); Pasteurella, Simkania (BCG non-durable responders)	BC vs. adjacent non-cancerous; BCG post-therapy recurrence analysis
Knorr et al. (49)	2024	11 (urine); 57 (10 fresh tissue, 23 FFPE BCG responders, 24 FFPE BCG non-responders)		Urine Tissue	Different	Different	NR	Lactobacillus gasseri, Lactobacillus johnsonii (BCG Responders); Corynebacterium kroppenstedtii, Streptococcus spp. (BCG Non-Responders)	BCG post-therapy recurrence analysis

### 3.1 Urinary microbiota and BC

Studies on the urinary microbiome in BC patients have revealed significant alterations in microbial diversity and composition. However, findings across studies remain inconsistent. Popovic et al. and Wu et al. analyzed urine samples using 16S rRNA sequencing but reported differing results. Popovic et al. observed no significant differences in microbial diversity or abundance between BC patients and healthy controls, while Wu et al. reported higher alpha diversity in BC patients, noting differences at the genus level (36, 37). Wu et al. specifically identified higher Shannon and Simpson diversity indices in BC patients compared to controls, with *Streptococcus* and *Escherichia-Shigella* as dominant taxa (36, 37). Bi et al. found reduced abundances of Bifidobacterium and Lactobacillus in BC patients and identified Actinomyces europaeus as a potential biomarker for the disease (38). In contrast, Chipollini et al. reported reduced alpha diversity in BC patients, whereas Zeng et al. found alpha diversity significantly higher in BC patients, with a 145% increase in the Chao1 index and a 123% increase in the Ace index compared to controls (35, 39). Hussein et al. further supported the reduction of *Lactobacillus* abundance in urine of BC patients (40). These discrepancies may arise from variations in sequencing platforms, data analysis strategies, sample types (urine vs. tissue), and patient characteristics such as tumor stage and treatment history. Additionally, the urinary microbiome is highly influenced by external factors, including diet and antibiotic use, which may not be uniformly controlled across studies. To better elucidate the role of the urinary microbiome in BC, future research should incorporate larger cohorts and standardized methodologies for sample collection and data analysis.

### 3.2 Tissue-resident microbiota and BC

Changes in the microbiota within BC tissues are closely linked to the TME. Liu et al. performed 16S rRNA sequencing on 22 cancerous and 12 adjacent normal tissues, reporting significantly lower alpha diversity in cancer tissues, indicating reduced microbial diversity in BC (41). Mansour et al. compared microbiota in 10 catheter urine samples and 14 TURBT-resected tumor tissues, finding significantly higher abundances of *Akkermansia*, *Bacteroides*, *Clostridium*, *Enterobacter*, and *Klebsiella* in cancer tissues, while *Staphylococcus* and *Lactobacillus* were consistently present in both sample types (42). Further studies corroborated these findings. Parra-Grande et al. observed lower microbial richness in tumor tissues compared to paired non-tumor tissues, alongside higher *Actinobacteria* levels in non-tumor samples, supporting its potential protective role against BC (43). Similarly, Pederzoli et al. identified *Klebsiella* as more prevalent in the urine of female BC patients, while *Burkholderia* was enriched in cancer tissues (44). Yao et al., using RNA sequencing, highlighted the enrichment of *Pseudomonas*, *Porphyrobacter*, and *Acinetobacter* in cancer tissues, suggesting their potential involvement in BC progression (45). While multiple studies consistently report a reduction in microbial diversity within BC tissues compared to normal tissues. These variations may stem from differences in sample types (tumor tissue vs. urine), patient demographics (gender, age, lifestyle), and analytical approaches (16S rRNA sequencing vs. metagenomic sequencing). Additionally, the microbial composition may undergo dynamic changes at different stages of BC. Therefore, future longitudinal studies with standardized methodologies are essential to elucidate the evolving role of microbiota in BC development and progression.

### 3.3 Microbes associated with BC progression and recurrence

Alterations in the bladder microbiome are closely associated with BC progression and recurrence, with distinct microbial patterns influencing disease outcomes and therapeutic responses. Oresta et al. reported significant increases in *Veillonella* and *Corynebacterium* and a reduction in *Ruminococcus* in urine samples from BC patients, with these shifts correlating with disease advancement (46). Sun et al., using 2bRAD-M sequencing, found that NMIBC tissues exhibited higher microbial diversity than muscle-invasive bladder cancer (MIBC) tissues, with *Ralstonia* sp. dominating in MIBC, contrasting with *Acinetobacter guillouiae* and *Anoxybacillus rupiensis* in NMIBC (47). High-grade tumors were linked to reduced microbial diversity and richness. Bilski et al. reported lower Chao1 and Shannon indices in high-grade tumors compared to low-grade tumors, with notable sex-related differences in microbial composition at the phylum level (e.g., *Firmicutes* dominance in males, *Proteobacteria* in females) (48).

In recurrence, specific microbial patterns also emerged. Yao et al. identified higher levels of *Mycolicibacterium* and *Streptomyces* in patients with sustained responses to BCG therapy, while Knorr et al. found increased *Lactobacillus* levels in BCG responders, suggesting protective effects of these genera (45, 49). Conversely, *Micrococcus* and *Brachybacterium* were enriched in recurrent patients (39). Hussein et al. observed post-TURBT increases in *Veillonella* and *Bifidobacterium* in recurrent cases, while *Escherichia-Shigella* and *Helococcus* were more abundant in non-recurrent cases (50). Qiu et al. linked higher alpha diversity in recurrent patients with elevated abundances of *Pseudomonas*, *Corynebacterium*, and *Acinetobacter*, potentially facilitating immune evasion and tumor growth (51). Particular attention should be given to BCG-refractory tumors, where up to 30-50% of NMIBC patients fail to respond to BCG intravesical therapy, leading to higher recurrence and progression rates (52–54). Microbial signatures in these cases often show enrichment of certain taxa associated with poor response (e.g., reduced Lactobacillus or altered diversity), which may impair BCG-induced immune activation by promoting an immunosuppressive TME (40, 55, 56). Recent studies suggest that gut or urinary microbiota modulation (e.g., via probiotics or potential fecal microbiota transplantation in preclinical models) could influence BCG responsiveness by enhancing Th1 responses and modulating immune cell infiltration (57, 58). Mechanisms involve altered TLR signaling and cytokine profiles, highlighting the need for microbiome-based predictors of BCG failure to guide alternative therapies like radical cystectomy or ICIs (59).

### 3.4 Gut microbiota and BC

Emerging evidence indicates that the gut microbiota exerts distal effects on bladder carcinogenesis through systemic immune modulation and metabolic signaling—a concept known as the “gut–bladder axis.” A case-control study in Harbin indicated that BC patients exhibited a significant reduction in gut microbiota diversity, with a notable decrease in the abundance of *Clostridium cluster XI* and *Prevotella*. This reduction was closely associated with low fruit intake among BC patients, and a significant decrease in butyrate concentration in their feces was also observed (60). Butyrate, a crucial short-chain fatty acid, plays a vital role in anti-inflammatory processes and in protecting the intestinal barrier. Its reduction may increase intestinal permeability, leading to chronic inflammation induced by elevated levels of LPS and D-lactic acid, thereby accelerating the development of BC. Furthermore, evidence from Mendelian randomization studies indicates a significant causal relationship between specific gut microbiota, such as *Bilophila*, and BC. This may occur through the modulation of amino acid and NAD metabolism pathways, promoting the onset of BC (61). Other microbiota, such as *Bifidobacterium* and *Actinobacteria*, are also associated with an increased risk of BC, while *Allisonella* has been found to be linked with a reduced risk of both bladder and prostate cancers (62). These microbiota alterations may influence tumorigenesis by modulating host metabolic pathways, immune signaling, and inflammatory responses. In the field of cancer immunotherapy, further research has uncovered the role of gut microbiota in regulating the therapeutic efficacy of BC treatments. The abundance of *Parabacteroides distasonis* was significantly higher in healthy controls than in BC patients. This bacterium enhances the infiltration of CD4+ and CD8+ T cells within tumors and activates anti-tumor immune pathways, thereby significantly improving the efficacy of anti-PD-1 treatment (63). This finding suggests that specific gut microbiota could serve as a potential adjunct to immunotherapy.

## 4 Mechanistic interplay: microbes, immunosenescence, and TME

### 4.1 Microbial modulation of epithelial integrity and EMT activation

#### 4.1.1 Microbial dysbiosis and EMT activation

Recent bioinformatics studies analyzing the microbial communities in BC have identified strong associations between specific microorganisms and the expression of EMT-related genes. Specifically, an analysis of tumor samples from over 400 patients with MIBC revealed significant correlations between various microorganisms, including *E. coli*, butyrate-producing bacterium SM4/1, and an *Oscillatoria* species, and the expression of classic EMT-related genes such as E-cadherin, vimentin, snail family transcriptional repressor 2 (SNAI2), snail family transcriptional repressor 3 (SNAI3), and twist family BHLH transcription factor 1 (TWIST1). Additionally, the study uncovered significant links between these microorganisms and the expression of extracellular matrix (ECM)-related genes, particularly those encoding collagen and elastin. These findings suggest that intratumoral microbiota may influence EMT and, consequently, clinical outcomes in BC (64). However, it is crucial to emphasize that these results are derived primarily from correlation-based bioinformatics analyses, and a direct causal relationship between microbial presence and EMT gene regulation remains to be established. While these associations provide compelling evidence for a potential microbial role in tumor progression, mechanistic insights into how these bacteria modulate EMT in BC are still lacking and require experimental validation.

Moreover, intratumoral bacteria are not randomly distributed but are highly organized within distinct ecological niches that are often characterized by immunosuppressive conditions and poor vascularization. By remodeling the TME and promoting cellular heterogeneity, bacteria may exert profound effects on tumor progression (65). Bacterial invasion can induce the upregulation of genes associated with inflammation, EMT, hypoxia response, and DNA repair, leading to the emergence of distinct cancer cell subpopulations with enhanced invasive potential. For example, in colorectal cancer (CRC), *Fusobacterium nucleatum* infection has been shown to drive the transition from collective migration to single-cell invasion, with significant activation of tumor progression signaling pathways (65). While these observations have been experimentally validated in CRC, direct evidence supporting a similar bacterial-driven EMT mechanism in BC is currently lacking. Thus, although bioinformatics analyses provide valuable insights into potential microbial contributions to EMT and tumor progression pathways, further in-depth mechanistic studies are necessary to confirm the direct role of specific bacteria in BC pathogenesis.

#### 4.1.2 Bacterial ECM degradation and remodeling

In BC, bacteria residing within the tumor stroma may influence extracellular matrix (ECM) integrity through the secretion of proteolytic enzymes. Bacterial proteases such as collagenase, elastase, and alkaline protease are capable of degrading key ECM components including collagen and elastin, thereby weakening structural barriers and potentially facilitating bacterial persistence (66, 67). Among these enzymes, collagenases are particularly relevant due to their broad substrate specificity and their ability to disrupt intercellular junctions and tissue organization (68–70).

Additionally, several Gram-positive bacteria produce hyaluronidase, an enzyme that hydrolyzes hyaluronic acid (HA), a major ECM component involved in tissue cohesion and cell adhesion. The degradation of HA provides nutrients for bacterial metabolism and may enhance tissue permeability, conditions that could theoretically support local invasion (71).

**Figure 2:**
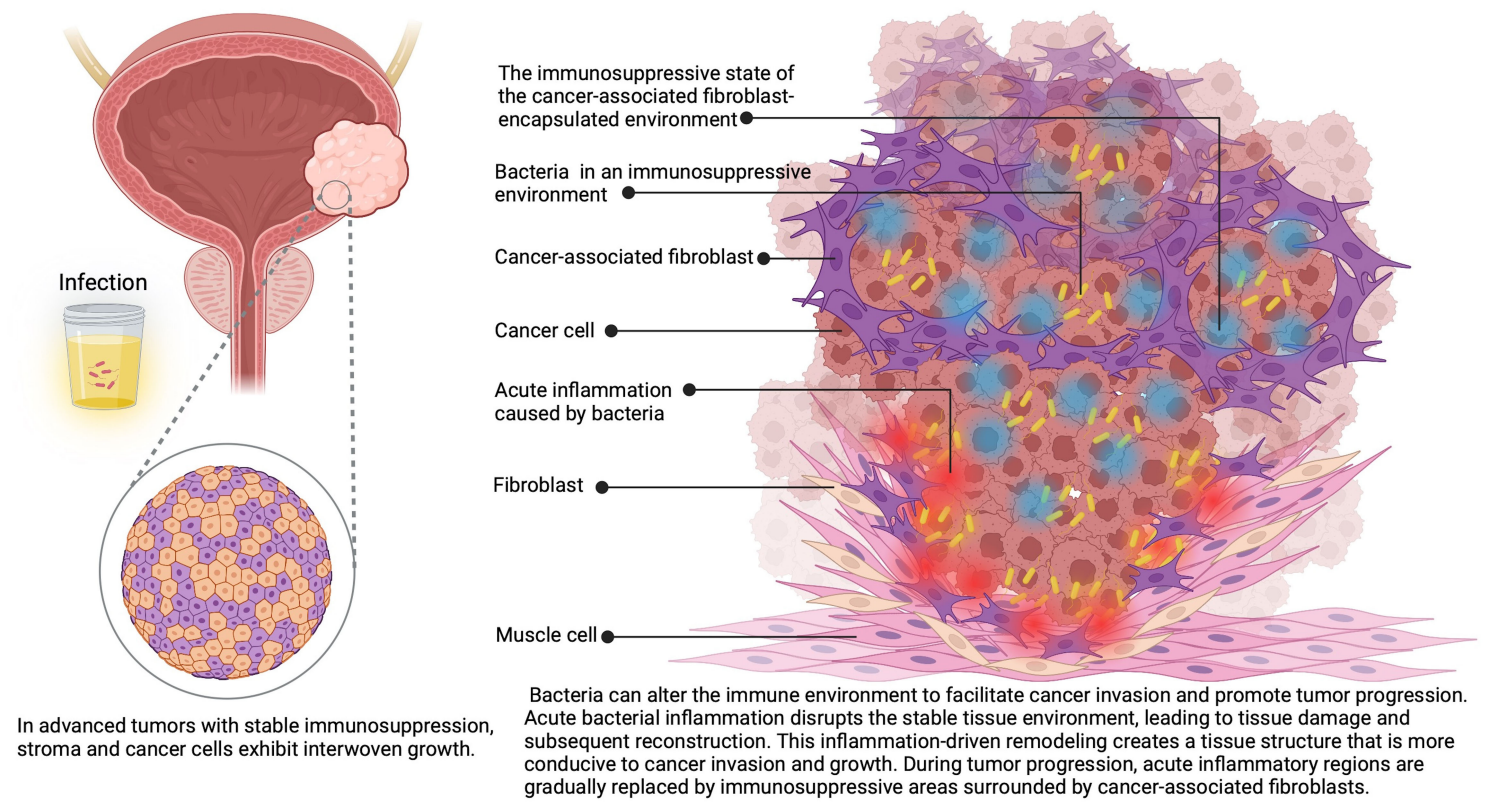
In progressive bladder cancer, cancer cells and CAFs grow intertwined. CAFs create an immunosuppressive environment that facilitates the rapid proliferation of cancer cells. Along with inflammation induced by bacterial infections, they manipulate and exploit the immune response, synergistically driving rapid cancer progression.

Beyond structural effects, bacterial proteases have been implicated in modulating host immune signaling in other pathological contexts by degrading cytokines and growth factors (72, 73). However, direct evidence of such mechanisms in BC remains scarce. While these findings collectively suggest that bacterial proteolytic activity might contribute to ECM remodeling within the bladder microenvironment (**Figure 2**), further mechanistic studies are needed to confirm its direct role in tumor progression and immune modulation in BC.

#### 4.1.3 Post-translational modulation by microbes

In addition to enzymatic ECM degradation, bacteria can also induce post-translational modifications (PTM) of host proteins, altering the biochemical and physical properties of the TME. For instance, *Porphyromonas gingivalis*, a known periodontal pathogen, produces peptidylarginine deiminase, an enzyme responsible for citrullination of collagen type I, which can disrupt the interactions between fibroblasts and collagen fibers (74, 75). These modifications may alter ECM stiffness and architecture, further promoting cancer cell invasion and metastasis. However, the functional significance of such bacterial-driven ECM modifications in BC and other solid tumors remains unclear. Although PTM-induced ECM alterations have been observed in multiple cancer types, their specific contribution to BC progression is yet to be fully elucidated and requires further experimental investigation.

### 4.2 Immunosenescence: the aging immune system and infection susceptibility

Immunosenescence—the progressive decline of immune function associated with aging—represents a critical but underappreciated determinant of host susceptibility to infection-driven carcinogenesis. In the context of bladder cancer (BC), recurrent microbial exposure and chronic urinary tract infections act as persistent antigenic stimuli that accelerate immune exhaustion and cellular senescence. This infection-induced immune aging not only impairs pathogen clearance but also fosters a permissive microenvironment that supports tumor initiation and progression (76, 77). Therefore, immunosenescence should not be viewed solely as an age-related phenomenon but as an integral component of the infection–microbiota–tumor axis.

#### 4.2.1 Immune aging in the TME

Such senescent immune phenotypes are further aggravated by recurrent infections, particularly in the bladder where chronic bacterial exposure induces continuous antigenic stimulation. This suggests that infection not only coexists with immune aging but actively accelerates it, shaping a tumor-permissive environment. Senescence affects both innate and adaptive immune cells, compromising their surveillance, cytotoxicity, and signaling capacity. CD8^+^ T cells exhibit classic immunosenescent phenotypes: loss of CD28, upregulation of KLRG1 and CD57, and metabolic dysregulation involving oxidative phosphorylation, ROS accumulation, and mitochondrial remodeling (78–86). Unlike exhausted T cells, senescent T cells are metabolically active but irreversibly dysfunctional, limiting the efficacy of checkpoint blockade therapies (87, 88). Thymic involution further restricts naïve T cell output, exacerbating immune decline. Clinical and preclinical studies have shown that the TME directly induces immunosenescence in tumor-infiltrating lymphocytes. For instance, CD8^+^ T cells in breast cancer brain metastases lose migratory and cytotoxic function despite originating from healthy lymphoid niches (78, 89).

NK cells and myeloid compartments are similarly affected. NK cells in the elderly show altered subset distributions (e.g., CD56dim accumulation), reduced activating receptor expression, and metabolic rewiring induced by tumor-secreted cAMP (90–92). Aging macrophages often display reduced antigen presentation and altered Toll-like receptor (TLR) signaling; however, their polarization state appears context-dependent. Many studies report a shift toward M2-like, pro-tumor phenotypes that reinforce immunosuppression and angiogenesis (93–101). However, others have documented sustained or even heightened pro-inflammatory M1-like activity associated with systemic “inflammaging” (102, 103). This bidirectional plasticity underscores the complexity of macrophage aging within the TME. Regarding BCG therapy in BC, which promotes macrophage repolarization from M2 (immunosuppressive) to M1 (pro-inflammatory) phenotypes to enhance anti-tumor immunity (104, 105), comparative analyses with other cancers reveal limitations. In colorectal and lung cancers, M1 macrophages can paradoxically promote tumor progression by secreting pro-angiogenic factors or fostering chronic inflammation, leading to mixed M1/M2 states that support metastasis (106). Unlike BC, where BCG-induced M1 shifts are often beneficial in NMIBC, these approaches have been less successful in solid tumors like melanoma, where M1 activation may exacerbate TME heterogeneity and resistance (107, 108). This highlights the tumor-specific context of macrophage reprogramming and the need for targeted strategies to avoid unintended pro-tumor effects. Senescent neutrophils contribute to tumor progression through the senescence-associated secretory phenotype (SASP), promoting myeloid-derived suppressor cells (MDSCs) recruitment and ECM remodeling. Markers such as TREM2 and CXCR4+CD62L^low phenotypes are associated with metastasis and therapeutic resistance (109–115). In addition, senescent dendritic cells, often modulated by tumor-derived γδ regulatory T cells (Tregs), suppress effector T cell differentiation via PD-L1 and STAT3 signaling (116).

#### 4.2.2 Impact on tumor surveillance and chronic inflammation

Infection acts as both a trigger and an amplifier of immunosenescence. Recurrent bacterial colonization, especially by uropathogens, perpetuates inflammatory signaling that exhausts immune competence. Immunosenescence also impacts systemic immune equilibrium, particularly mucosal immunity and microbial recognition. B cell senescence manifests as reduced bone marrow output, metabolic inflammation, and impaired antibody production (117–121). This state not only affects pathogen clearance but also contributes to T cell dysfunction via pro-inflammatory cytokines and clonal restriction of the T cell receptor repertoire (119). These immune alterations collectively diminish host capacity to eliminate pathogens, creating a permissive environment for microbial colonization and chronic infection. In the bladder, age-related immune decline may promote the persistence of uropathogens and delay resolution of UTIs, especially in elderly patients. This in turn can lead to prolonged inflammation, epithelial damage, and microbial-driven carcinogenesis. From a translational perspective, immunosenescence represents a potential therapeutic target. Rejuvenation strategies, such as senolytic therapies, metabolic reprogramming, and targeted epigenetic modulation, may help restore immune competence in elderly patients and improve response to both immunotherapy and microbiome-modulating interventions. Thus, age-related immune decline and infection-induced inflammation converge to form a pathogenic feedback loop that sustains microbial persistence and tumor-promoting chronic inflammation in the bladder.

### 4.3 Immune reprogramming in response to microbial infections

#### 4.3.1 Uropathogenic E. coli and host immune modulation

**Figure 3:**
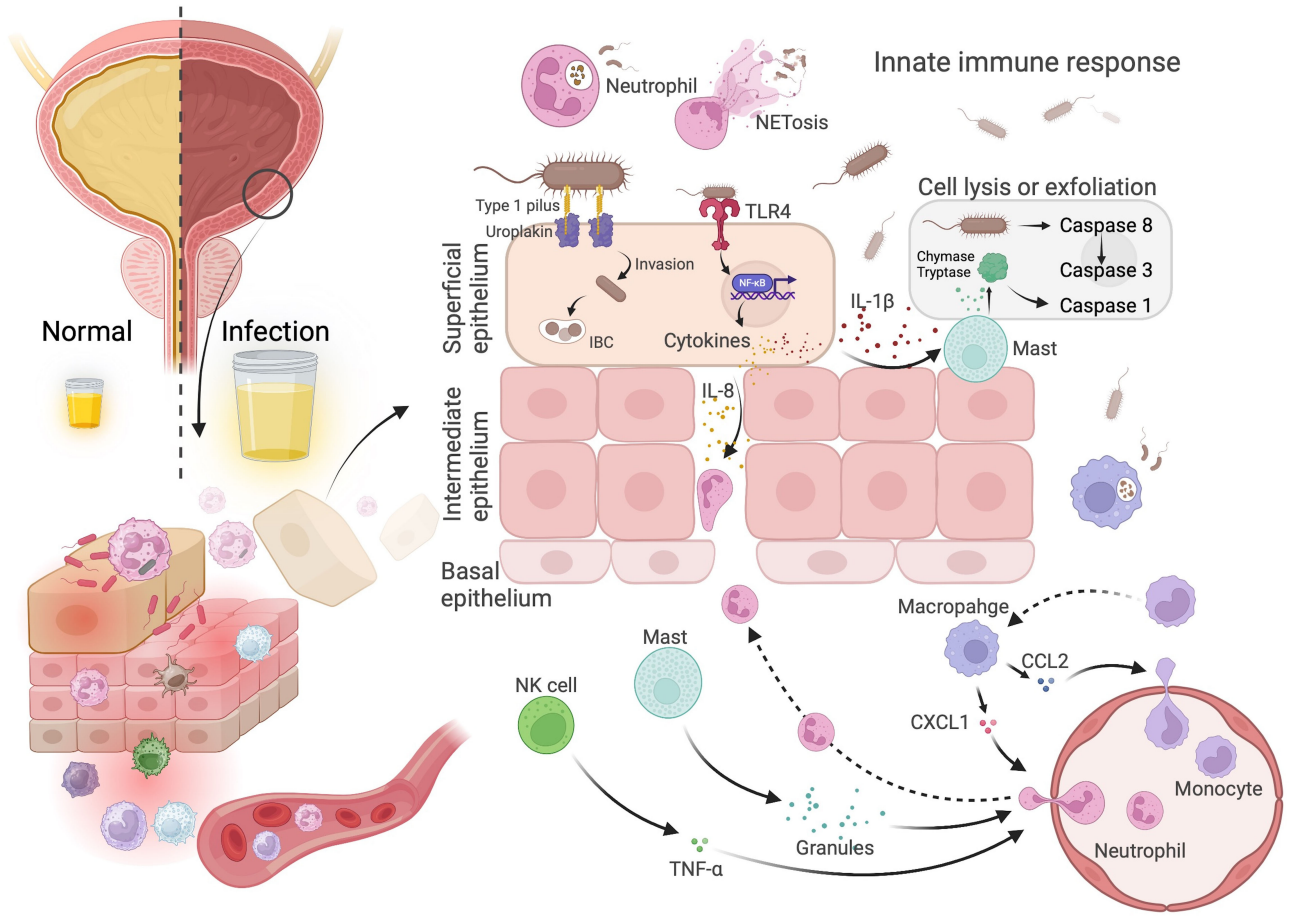
Bladder infections caused by *E. coli* activate the innate immune response. Immune cells are recruited to the infection site to capture and clear *E. coli*. Neutrophils and macrophages eliminate *E. coli* through phagocytosis, while mast cells facilitate the shedding and death of epithelial cells infected by *E. coli*. *E. coli* can evade the acute inflammatory response by invading and colonizing host cells.

The innate immune system promptly detects uropathogenic Escherichia coli (UPEC) through pattern recognition receptors (PRRs), particularly Toll-like receptor 4 (TLR4), which recognizes bacterial LPS. Activation of TLR4 initiates the NF-κB signaling pathway and stimulates the release of pro-inflammatory cytokines such as IL-6, IL-8, and TNF-α, which recruit neutrophils and macrophages (122–125). These immune cells utilize phagocytosis, ROS, and neutrophil extracellular traps (NETs) to eliminate bacterial invaders (126–129). Macrophages further contribute to immune activation through secretion of chemokines such as CXCL1 and CCL2, while dendritic cells (DCs) function as antigen-presenting cells that activate adaptive immune responses by priming T cells (130–134). Interestingly, bacterial products may also exert anti-tumor effects. For instance, E. coli supernatants downregulate c-MYC and pro-inflammatory cytokines like IL-1β and CCL2, while upregulating NQO1 expression. These changes promote apoptosis via BAX activation and suppression of anti-apoptotic BCL2 in bladder cancer cells (135–138). However, while bacterial-induced apoptosis may initially appear beneficial, it can also modulate the TME in ways that support immune evasion or chronic inflammation. UPEC adheres to bladder epithelial cells via type 1 pili and FimH adhesin, initiating actin reorganization and PI3-kinase signaling to promote bacterial internalization (139–143) (**Figure 3**). The formation of intracellular bacterial communities (IBCs) enables UPEC to evade host immunity, persist intracellularly, and drive chronic inflammation—factors that may contribute to bladder cancer progression.

#### 4.3.2 Infection-induced epithelial exfoliation and barrier disruption

Bladder epithelial cell exfoliation is a key host defense strategy against UTI. Upon E. coli infection, bladder epithelial cells secrete IL-1β, which recruits mast cells (MCs) to the infection site. These MCs release granules rich in chymase and tryptase, triggering apoptosis and exfoliation of infected cells (144–147). This response reduces bacterial burden and promotes epithelial renewal. Notably, mast cells undergo functional switching from a pro-inflammatory to an anti-inflammatory state approximately six hours post-infection, facilitating immune resolution and tissue repair (146, 148). UPEC-derived virulence factors, such as hemolysin, may further contribute to epithelial disruption (140, 149). While exfoliation is essential for bacterial clearance, it may also expose basal epithelial cells to inflammation-induced stress, potentially initiating or exacerbating malignant transformation.

### 4.4 Tumor susceptibility to bacterial invasion

#### 4.4.1 Morphological and barrier defects

BC cells exhibit significant morphological alterations compared to normal urothelial cells, which enhance their susceptibility to bacterial infection. Normal urothelial cells form a well-organized epithelial barrier with tight and adherens junctions, preventing bacterial adherence and protecting against infection (150–152). These cells are typically flat, multilayered, and exhibit apical-basal polarity, with smooth membranes and limited adhesion sites. Tight junction proteins, such as Occludin, Claudins, and E-cadherin, play a crucial role in maintaining this barrier function (153–155).

**Figure 4:**
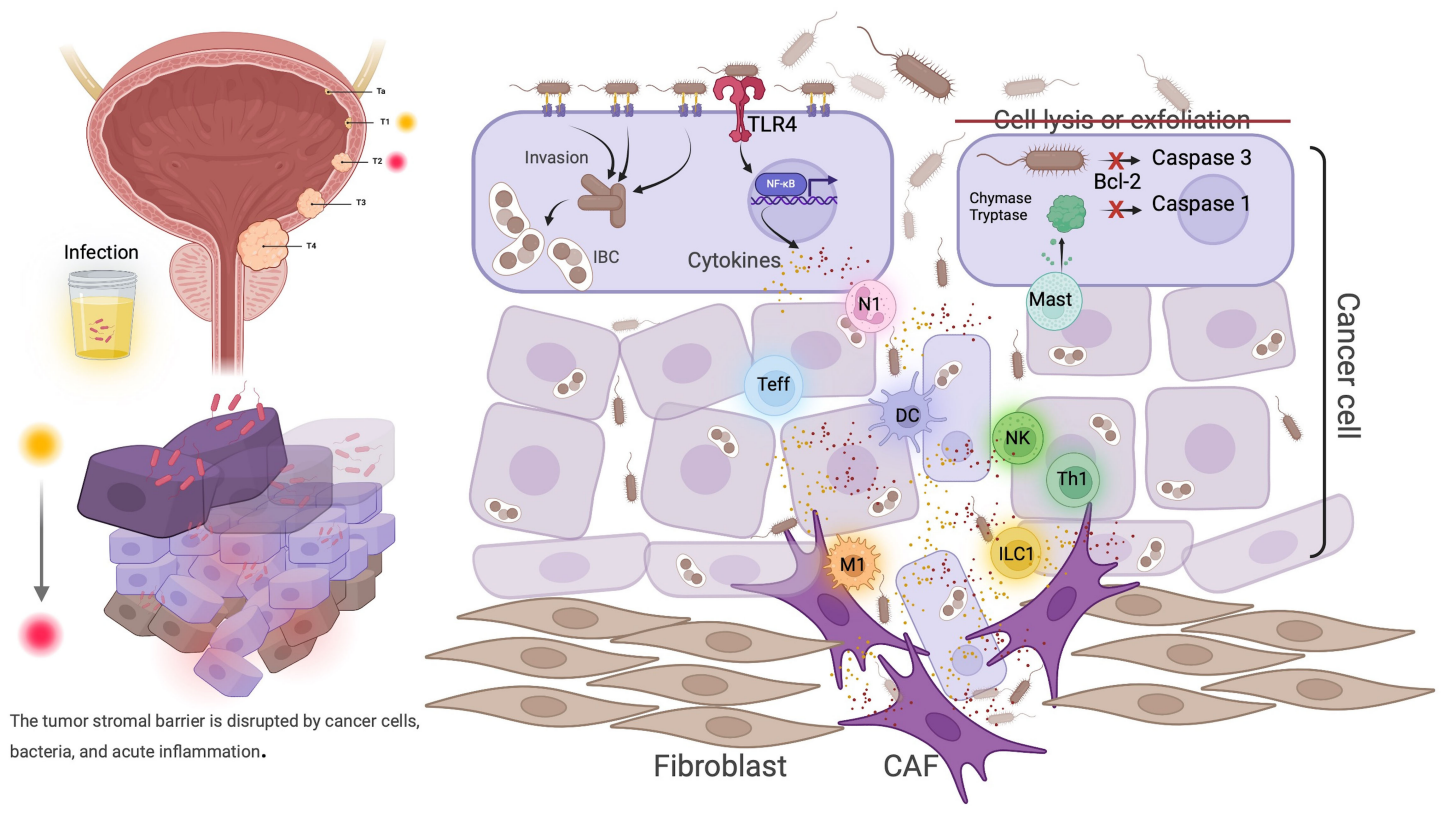
In superficial bladder cancer, acute inflammatory responses triggered by bacterial infections can significantly promote cancer progression. While acute inflammation can inhibit and kill cancer cells, cancer cells differ from normal urothelial cells by upregulating anti-apoptotic genes such as BCL2, thereby suppressing apoptosis. Infected cancer cells are less prone to detachment or death, exhibit morphological changes, and have disrupted intercellular connections, leading to widespread invasion and intracellular bacterial colonization. The deep invasion of acute inflammation can cause substantial damage to the stroma. Cancer cells are more prone to invasion and growth, and can drive the malignant transformation of fibroblasts through inflammation.

In contrast, bladder cancer cells display surface microvilli proliferation, pseudopod formation, and altered glycosylation patterns, including the abnormal expression of Tn and sialyl-Tn antigens, which provide additional bacterial adhesion sites (156, 157). Loss of cellular polarity and weakened intercellular junctions result in larger gaps between cells, exposing extracellular matrix components like fibronectin and laminin, which serve as binding sites for bacterial adhesion (158). Moreover, bladder cancer cells often resist infection-induced apoptosis, partly due to increased BCL2 expression, which allows them to persist in inflammatory environments triggered by infection, further promoting tumor proliferation and invasion (**Figure 4**).

Clinically, these morphological changes contribute to the increased risk of UTIs in BC patients, with bacterial adhesion exacerbating disease progression. Targeting bacterial adhesion, for instance by inhibiting lectin-glycan interactions, may reduce colonization. These altered morphological features could also serve as biomarkers for assessing infection risk in BC.

#### 4.4.2 Immunosuppressive tumor states

The immunosuppressive microenvironment in bladder cancer facilitates bacterial colonization by dampening the host’s immune response. In healthy urothelial tissue, antimicrobial peptides like β-defensins, along with cytokines such as IL-6 and IL-8, are secreted to recruit immune cells and combat pathogens (159–162). However, BC cells often secrete immunosuppressive cytokines, including TGF-β and IL-10, which impair immune cell activity and local immune responses (163–166). Treg infiltration further diminishes effector T cell function, hindering the clearance of pathogens and tumor cells (167).

BC cells also frequently downregulate MHC class I expression, reducing antigen presentation and immune recognition of both bacterial pathogens and tumor cells (168, 169). This creates an immunosuppressive TME that favors bacterial persistence. Studies have shown that bacterial presence in BC tissues correlates with increased infiltration of CD66b+ neutrophils and higher levels of immunosuppressive molecules like ARG1 and CTLA4. Additionally, activation of mitogen-activated protein kinase (MAPK) signaling in this context supports chronic bacterial colonization and inflammation, potentially accelerating tumor progression (65).

Clinically, targeting immunosuppressive pathways—such as blocking ARG1 or CTLA4—could enhance the immune system’s ability to clear both bacterial infections and cancer cells. Moreover, microbiome-based interventions, such as probiotics or engineered bacteria, may help modulate the TME and reduce bacterial infections, offering a potential complement to conventional cancer therapies.

### 4.5 Gut microbiota and immunosenescence

Immunosenescence, the gradual decline in immune system function during aging, is closely associated with chronic inflammation (inflammaging) and the onset of age-related diseases, such as cancer and infections. The gut microbiota plays a central role in this process. With aging, the composition and functionality of the gut microbiota undergo significant changes, manifested as a reduction in diversity, a decrease in beneficial bacteria (e.g., *Faecalibacterium prausnitzii* and *Akkermansia muciniphila*), and an increase in pro-inflammatory bacteria (e.g., *Enterobacteriaceae* and *Proteobacteria*) (170). These changes are closely related to T-cell aging, especially in middle-aged and older individuals. Individuals with lower gut microbiota richness show significantly higher mRNA biomarkers of T-cell senescence, and Shannon diversity is negatively correlated with the epigenetic age of T-cell DNA methylation (171). Additionally, metagenomic analysis of centenarians (≥90 years) revealed that age-related changes in the gut microbiota, chronic inflammation, and microbial metabolic reprogramming are key drivers of immunosenescence (172). These alterations in the gut microbiota accelerate the process of immune aging through various mechanisms. This process is accompanied by functional decline in specific immune cells, such as long-term stimulation by symbiotic bacteria that leads to the proliferation arrest and aging of germinal center (GC) B cells in Peyer’s patches (PPs), resulting in the loss of PP function. This damage, through bacterial-dependent compensatory mechanisms, promotes further aging of B cells in lymphoid follicles (ILFs), ultimately leading to a significant reduction in IgA production and diversity. This weakens the regulation of the gut microbiota, creating a vicious cycle between gut microbiota and immunosenescence (173).

Interventions targeting the gut microbiota are considered an important strategy to delay immunosenescence. For example, probiotics, such as *Lactobacillus plantarum*, can restore aging-related dendritic cell function and improve gut immune regulation in elderly individuals (174). Lifestyle interventions, such as caloric restriction, can reshape the gut microbiota and promote the functional recovery of T cells and B cells, thereby mitigating immunosenescence (175). Furthermore, fecal microbiota transplantation and combined interventions with prebiotics and probiotics have been shown to improve the composition of the gut microbiota in elderly patients, enhancing their response to immune checkpoint blockade (ICB) therapy, which provides anti-tumor benefits (176). Studies have also demonstrated that ICB therapy is associated with the enrichment of specific microbiota, particularly in elderly patients. The modulation of age-related gut microbiota is considered a key factor in improving ICB efficacy (177).

## 5 Therapeutic implications and future perspectives

The future of BC treatment lies in innovative, integrated strategies that combine microbiome modulation and immune therapies. These approaches focus on overcoming challenges such as microbial dysbiosis and immunosenescence, leveraging cutting-edge technologies like artificial intelligence (AI) and CRISPR for precision medicine. The integration of gut–bladder axis interventions with microbiome–immune therapies provide new avenues to improve clinical outcomes for BC patients. This chapter explores three major therapeutic strategies that hold great promise in advancing BC treatment.

### 5.1 Personalized microbiota modulation

Microbiome alterations in BC present a unique opportunity for personalized treatment strategies. Dynamic changes in microbial communities, such as shifts in urinary or stool microbiota, offer potential biomarkers for tumor progression and therapeutic response. Non-invasive microbiome analyses can enable real-time monitoring, guiding therapeutic interventions. For instance, machine learning algorithms can identify specific taxa like *Lactobacillus* or *Veillonella*, which are associated with either tumor progression or favorable responses to immunotherapy. Additionally, CRISPR-based microbial engineering could be harnessed to generate beneficial metabolites like short-chain fatty acids (SCFAs) or indole derivatives, potentially enhancing immune responses and improving the TME. Personalized microbiome modulation could significantly optimize treatment efficacy and patient outcomes (178).

### 5.2 Microbiome–immunotherapy combinations

The combination of microbiome modulation with immunotherapies represents a highly promising therapeutic strategy. Recent studies have shown that prebiotics, synbiotics, and microbiota-derived metabolites, such as butyrate, can work synergistically with immune checkpoint inhibitors (ICIs). These compounds enhance tumor-infiltrating lymphocyte (TIL) activity and modulate immunosuppressive cells like MDSCs and Tregs. Additionally, engineered microbial therapeutics that secrete immune-activating molecules (e.g., IL-15, GM-CSF) or anti-inflammatory agents (e.g., IL-10) could help reverse immunosenescence, particularly in aging populations. Preclinical studies have shown that *Lactobacillus reuteri* can translocate into tumors and release the tryptophan metabolite indole-3-aldehyde (I3A), which activates the aryl hydrocarbon receptor in CD8^+^ T cells and enhances anti-PD-L1 immune checkpoint blockade efficacy in melanoma models (179). Additionally, engineered bacterial platforms releasing PD-L1 nanobodies have demonstrated tumor-localized immune activation and reduced systemic toxicity in breast cancer models, highlighting the potential of microbiome-based strategies to modulate the TME and improve immunotherapy outcomes (180). While these findings suggest a promising direction for microbiome engineering in cancer therapy, comparable work specific to bladder cancer models has not yet been reported. This combination approach has the potential to enhance immune responses during various stages of treatment, from reducing preoperative infection risks to boosting immune memory during ICI therapy. Future research will focus on optimizing microbiome–immunotherapy interactions and tailoring these approaches to individual patient needs.

### 5.3 Gut–bladder axis therapeutic targeting

The interaction between the gut and bladder microbiota plays a crucial yet underexplored role in BC progression. Gut-derived metabolites, such as SCFAs and indoles, influence bladder immunity through systemic circulation and can modulate the bladder’s immune microenvironment. Targeting this “gut-bladder axis” with therapeutic interventions could offer a novel approach to improving the TME. For example, dietary modifications, such as high-fiber diets, or probiotic formulations that promote butyrate production, could boost systemic T-cell regeneration and enhance TIL activity within the bladder. These strategies could potentially reduce chronic inflammation, improve antigen presentation, and modulate the immune responses in the bladder, ultimately contributing to better treatment outcomes (178).

### 5.4 Multi-modal microbiome therapies

Future BC treatments will likely involve multi-modal strategies that combine microbiome-based therapies with conventional cancer treatments. Personalized microbiome modulation can complement traditional therapies like chemotherapy and radiotherapy, as well as advanced treatments such as ICIs. Customized microbial formulations may also enhance post-surgical recovery, improve patients’ immune response, and reduce recurrence rates in high-risk individuals. Furthermore, microbiome interventions could be incorporated into tumor vaccines to boost long-term anti-tumor immunity. As microbiome data accumulation accelerates, AI will play a critical role in optimizing these multi-modal strategies by identifying microbial targets, predicting patient-specific responses, and enabling truly personalized therapies. Examples of enhanced BCG-based approaches in bladder cancer include the use of PD-1/PD-L1 inhibitors such as pembrolizumab, which as monotherapy has demonstrated complete response rates of ~40% in BCG-unresponsive NMIBC cohorts (181). Recombinant BCG strains engineered to express IL-15 fused with antigen 85B have shown enhanced immunogenicity and prolonged survival in preclinical mouse bladder cancer models, associated with increased neutrophil and chemokine responses (182). Additionally, BCG combined with chemotherapy agents such as gemcitabine is being evaluated in clinical settings, with some early studies reporting promising response rates, though results remain preliminary and variable (183).

## 6 Conclusion

The relationship between the microbiome and BC is increasingly recognized as a critical factor in tumor initiation, progression, and recurrence. Both urinary and gut microbiota have significant roles in shaping the TME, with microbial infections serving as key drivers of cancer development. Disruptions in microbial communities—microbial dysbiosis—are strongly associated with chronic inflammation, immune modulation, and alterations in urothelial integrity, all of which contribute to tumor progression and metastasis. Bacterial infections, particularly those involving uropathogenic species, exacerbate these processes, facilitating the initiation and invasiveness of BC.

Infection-induced mechanisms, including microbial dysbiosis, drive important changes in the immune landscape of the TME. These changes are often exacerbated by immunosenescence, which weakens immune surveillance and increases susceptibility to persistent infections. Immunosenescence, while not the primary driver of BC, enhances the persistence of microbial dysbiosis by impairing the body’s ability to mount effective immune responses against both pathogens and tumor cells. This immune decline creates a favorable environment for tumorigenesis, as chronic inflammation and microbial imbalance promote immune evasion and tumor progression.

Future research should focus on elucidating how the gut and urinary microbiota interact to influence BC, particularly through their impact on immune responses and TME remodeling. Understanding the mechanisms by which microbial dysbiosis influences the development of BC will be essential for the development of novel diagnostic and therapeutic approaches. Microbiome-based therapies, particularly those targeting microbial imbalances and combined with immune modulation, offer promising avenues for personalized BC treatment. A more comprehensive understanding of these interactions will not only advance our knowledge of BC pathogenesis but also provide new strategies for improving early detection, prognosis, and treatment outcomes.

## Glossary

APCs: antigen-presenting cells
ARG1: arginase 1
BAX: Bcl-2-associated X protein
BCL2: B-cell lymphoma 2
BCG: Bacillus Calmette-Guérin
BECs: bladder epithelial cells
CAFs: cancer-associated fibroblasts
CCL2: C-C motif chemokine ligand 2
CTLA4: cytotoxic T-lymphocyte–associated protein 4
CTLs: cytotoxic T lymphocytes
CX3CL1: C-X3-C motif chemokine ligand 1
CXCL12: C-X-C motif chemokine ligand 12
DCs: dendritic cells
DCA: deoxycholic acid
ECM: extracellular matrix
*E. coli*: Escherichia coli
EMT: epithelial–mesenchymal transition
FM: first-morning
FXR: farnesoid X receptor
IBCs: intracellular bacterial communities
IFN-γ: interferon-gamma
IL: interleukin
ILCs: innate lymphoid cells
LCA: lithocholic acid
LPS: lipopolysaccharide
MAPK: mitogen-activated protein kinase
MCP-1: monocyte chemoattractant protein-1
MCs: mast cells
MDSCs: myeloid-derived suppressor cells
MHC: major histocompatibility complex
MIBC: muscle-invasive bladder cancer
MMPs: matrix metalloproteinases
MNU: N-methyl-N-nitrosourea
NETs: neutrophil extracellular traps
NF-κB: nuclear factor kappa B
NLR: neutrophil-to-lymphocyte ratio
NMIBC: non-muscle-invasive bladder cancer
NK: natural killer
NO: nitric oxide
NQO1: NAD(P)H quinone dehydrogenase 1
PAMPs: pathogen-associated molecular patterns
PCR: polymerase chain reaction
PD-1: programmed cell death protein 1
PD-L1: programmed death-ligand 1
PI3-kinase: phosphoinositide 3-kinase
PRRs: pattern recognition receptors
ROS: reactive oxygen species
SCFAs: short-chain fatty acids
SNAI2: snail family transcriptional repressor 2
SNAI3: snail family transcriptional repressor 3
TANs: tumor-associated neutrophils
TAAs: tumor-associated antigens
TCA: tricarboxylic acid
TGF-β: transforming growth factor-beta
Th1: T helper type 1
Th2: T helper type 2
TLRs: toll-like receptors
Tregs: regulatory T cells
TURBT: transurethral resection of bladder tumor
Teff: effector CD8+ T cells
Tex: exhausted CD8+ T cells
TGR5: G-protein-coupled bile acid receptor 5
TNF-α: tumor necrosis factor-alpha
TWIST1: twist family BHLH transcription factor 1
UPEC: uropathogenic *Escherichia coli*
UTIs: urinary tract infections
UTP: uridine triphosphate
VEGF: vascular endothelial growth factor

## References

[1] Compérat E Amin MB Cathomas R Choudhury A De Santis M Kamat A . Current best practice for bladder cancer: a narrative review of diagnostics and treatments. Lancet. (2022) 400:1712–21. doi: 10.1016/s0140-6736(22)01188-6, PMID: 36174585

[2] Antoni S Ferlay J Soerjomataram I Znaor A Jemal A Bray F . Bladder cancer incidence and mortality: A global overview and recent trends. Eur Urol. (2017) 71:96–108. doi: 10.1016/j.eururo.2016.06.010, PMID: 27370177

[3] Siegel RL Miller KD Wagle NS Jemal A . Cancer statistics, 2023. CA Cancer J Clin. (2023) 73:17–48. doi: 10.3322/caac.21763, PMID: 36633525

[4] Burger M Catto JW Dalbagni G Grossman HB Herr H Karakiewicz P . Epidemiology and risk factors of urothelial bladder cancer. Eur Urol. (2013) 63:234–41. doi: 10.1016/j.eururo.2012.07.033, PMID: 22877502

[5] Mantica G Terrone C Der Merwe AV . Bladder cancer and associated risk factors: the african panorama. Eur Urol. (2021) 79:568–70. doi: 10.1016/j.eururo.2020.11.041, PMID: 33280932

[6] Honeycutt J Hammam O Fu CL Hsieh MH . Controversies and challenges in research on urogenital schistosomiasis-associated bladder cancer. Trends Parasitol. (2014) 30:324–32. doi: 10.1016/j.pt.2014.05.004, PMID: 24913983 PMC4085545

[7] Barsoum RS . Urinary schistosomiasis: review. J Adv Res. (2013) 4:453–9. doi: 10.1016/j.jare.2012.08.004, PMID: 25685452 PMC4293885

[8] El-Rifai W Kamel D Larramendy ML Shoman S Gad Y Baithun S . DNA copy number changes in Schistosoma-associated and non-Schistosoma-associated bladder cancer. Am J Pathol. (2000) 156:871–8. doi: 10.1016/s0002-9440(10)64956-5, PMID: 10702404 PMC1876852

[9] Pezone A Olivieri F Napoli MV Procopio A Avvedimento EV Gabrielli A . Inflammation and DNA damage: cause, effect or both. Nat Rev Rheumatol. (2023) 19:200–11. doi: 10.1038/s41584-022-00905-1, PMID: 36750681

[10] O’Byrne KJ Dalgleish AG Browning MJ Steward WP Harris AL . The relationship between angiogenesis and the immune response in carcinogenesis and the progression of Malignant disease. Eur J Cancer. (2000) 36:151–69. doi: 10.1016/s0959-8049(99)00241-5, PMID: 10741273

[11] Adebayo AS Suryavanshi MV Bhute S Agunloye AM Isokpehi RD Anumudu CI . The microbiome in urogenital schistosomiasis and induced bladder pathologies. PloS Negl Trop Dis. (2017) 11:e0005826. doi: 10.1371/journal.pntd.0005826, PMID: 28793309 PMC5565189

[12] Markowski MC Boorjian SA Burton JP Hahn NM Ingersoll MA Maleki Vareki S . The microbiome and genitourinary cancer: A collaborative review. Eur Urol. (2019) 75:637–46. doi: 10.1016/j.eururo.2018.12.043, PMID: 30655087 PMC9774685

[13] Gouveia MJ Santos J Brindley PJ Rinaldi G Lopes C Santos LL . Estrogen-like metabolites and DNA-adducts in urogenital schistosomiasis-associated bladder cancer. Cancer Lett. (2015) 359:226–32. doi: 10.1016/j.canlet.2015.01.018, PMID: 25615421

[14] Siegel RL Miller KD Jemal A . Cancer statistics, 2020. CA Cancer J Clin. (2020) 70:7–30. doi: 10.3322/caac.21590, PMID: 31912902

[15] Zeng G Zhu W Lam W Bayramgil A . Treatment of urinary tract infections in the old and fragile. World J Urol. (2020) 38:2709–20. doi: 10.1007/s00345-020-03159-2, PMID: 32221713

[16] Schaeffer AJ . Urinary tract infections in the elderly. Eur Urol. (1991) 19 Suppl:12–6. doi: 10.1159/000473669, PMID: 2022228

[17] El-Mosalamy H Salman TM Ashmawey AM Osama N . Role of chronic E. coli infection in the process of bladder cancer- an experimental study. Infect Agent Cancer. (2012) 7:19. doi: 10.1186/1750-9378-7-19, PMID: 22873280 PMC3511874

[18] Singh R Sharma G Priyadarshi S Fauzdar G . Prognostic significance of preoperative pyuria & neutrophil to lymphocyte ratio in patients with non-muscle-invasive bladder cancer: A prospective cohort study. Urologia. (2024) 91:69–75. doi: 10.1177/03915603231203780, PMID: 37909427

[19] Sazuka T Sakamoto S Imamura Y Nakamura K Yamamoto S Arai T . Relationship between post-void residual urine volume, preoperative pyuria and intravesical recurrence after transurethral resection of bladder carcinoma. Int J Urol. (2020) 27:1024–30. doi: 10.1111/iju.14352, PMID: 32875619

[20] Satake N Ohno Y Nakashima J Ohori M Tachibana M . Prognostic value of preoperative pyuria in patients with non-muscle-invasive bladder cancer. Int J Urol. (2015) 22:645–9. doi: 10.1111/iju.12788, PMID: 25912166

[21] Azuma T Nagase Y Oshi M . Pyuria predicts poor prognosis in patients with non-muscle-invasive bladder cancer. Clin Genitourin Cancer. (2013) 11:331–6. doi: 10.1016/j.clgc.2013.04.002, PMID: 23664207

[22] Redelman-Sidi G Glickman MS Bochner BH . The mechanism of action of BCG therapy for bladder cancer--a current perspective. Nat Rev Urol. (2014) 11:153–62. doi: 10.1038/nrurol.2014.15, PMID: 24492433

[23] Isali I Helstrom EK Uzzo N Lakshmanan A Nandwana D Valentine H . Current trends and challenges of microbiome research in bladder cancer. Curr Oncol Rep. (2024) 26:292–8. doi: 10.1007/s11912-024-01508-7, PMID: 38376627 PMC10920447

[24] Kawai K Yamamoto M Kameyama S Kawamata H Rademaker A Oyasu R . Enhancement of rat urinary bladder tumorigenesis by lipopolysaccharide-induced inflammation. Cancer Res. (1993) 53:5172–5., PMID: 8221653

[25] Jing W Wang G Cui Z Li X Zeng S Jiang X . Tumor-neutrophil cross talk orchestrates the tumor microenvironment to determine the bladder cancer progression. Proc Natl Acad Sci U.S.A. (2024) 121:e2312855121. doi: 10.1073/pnas.2312855121, PMID: 38713626 PMC11098120

[26] Abd-El-Raouf R Ouf SA Gabr MM Zakaria MM El-Yasergy KF Ali-El-Dein B . Escherichia coli foster bladder cancer cell line progression via epithelial mesenchymal transition, stemness and metabolic reprogramming. Sci Rep. (2020) 10:18024. doi: 10.1038/s41598-020-74390-5, PMID: 33093503 PMC7581527

[27] Nesi G Nobili S Cai T Caini S Santi R . Chronic inflammation in urothelial bladder cancer. Virchows Arch. (2015) 467:623–33. doi: 10.1007/s00428-015-1820-x, PMID: 26263854

[28] Russell SK Harrison JK Olson BS Lee HJ O’Brien VP Xing X . Uropathogenic Escherichia coli infection-induced epithelial trained immunity impacts urinary tract disease outcome. Nat Microbiol. (2023) 8:875–88. doi: 10.1038/s41564-023-01346-6, PMID: 37037942 PMC10159856

[29] Vermeulen SH Hanum N Grotenhuis AJ Castaño-Vinyals G van der Heijden AG Aben KK . Recurrent urinary tract infection and risk of bladder cancer in the Nijmegen bladder cancer study. Br J Cancer. (2015) 112:594–600. doi: 10.1038/bjc.2014.601, PMID: 25429525 PMC4453642

[30] Heidar NA Bhat TA Shabir U Hussein AA . The urinary microbiome and bladder cancer. Life (Basel). (2023) 13:812. doi: 10.3390/life13030812, PMID: 36983967 PMC10053959

[31] Russo AE Memon A Ahmed S . Bladder cancer and the urinary microbiome-new insights and future directions: A review. Clin Genitourin Cancer. (2024) 22:434–44. doi: 10.1016/j.clgc.2023.12.015, PMID: 38220540

[32] Friedrich V Choi HW . The urinary microbiome: role in bladder cancer and treatment. Diagnost (Basel). (2022) 12:2068. doi: 10.3390/diagnostics12092068, PMID: 36140470 PMC9497549

[33] Whiteside SA Razvi H Dave S Reid G Burton JP . The microbiome of the urinary tract--a role beyond infection. Nat Rev Urol. (2015) 12:81–90. doi: 10.1038/nrurol.2014.361, PMID: 25600098

[34] Hourigan SK Zhu W SWW W Clemency NC Provenzano M Vilboux T . Studying the urine microbiome in superficial bladder cancer: samples obtained by midstream voiding versus cystoscopy. BMC Urol. (2020) 20:5. doi: 10.1186/s12894-020-0576-z, PMID: 31992287 PMC6986141

[35] Chipollini J Wright JR Nwanosike H Kepler CY Batai K Lee BR . Characterization of urinary microbiome in patients with bladder cancer: Results from a single-institution, feasibility study. Urol Oncol. (2020) 38:615–21. doi: 10.1016/j.urolonc.2020.04.014, PMID: 32414567

[36] Bučević Popović V Šitum M Chow CT Chan LS Roje B Terzić J . The urinary microbiome associated with bladder cancer. Sci Rep. (2018) 8:12157. doi: 10.1038/s41598-018-29054-w, PMID: 30108246 PMC6092344

[37] Wu P Zhang G Zhao J Chen J Chen Y Huang W . Profiling the urinary microbiota in male patients with bladder cancer in China. Front Cell Infect Microbiol. (2018) 8167:167. doi: 10.3389/fcimb.2018.00167, PMID: 29904624 PMC5990618

[38] Bi H Tian Y Song C Li J Liu T Chen Z . Urinary microbiota - a potential biomarker and therapeutic target for bladder cancer. J Med Microbiol. (2019) 68:1471–8. doi: 10.1099/jmm.0.001058, PMID: 31418671

[39] Zeng J Zhang G Chen C Li K Wen Y Zhao J . Alterations in urobiome in patients with bladder cancer and implications for clinical outcome: A single-institution study. Front Cell Infect Microbiol. (2020) 10555508:555508. doi: 10.3389/fcimb.2020.555508, PMID: 33384966 PMC7769872

[40] Hussein AA Elsayed AS Durrani M Jing Z Iqbal U Gomez EC . Investigating the association between the urinary microbiome and bladder cancer: An exploratory study. Urol Oncol. (2021) 39:370. doi: 10.1016/j.urolonc.2020.12.011, PMID: 33436328

[41] Liu F Liu A Lu X Zhang Z Xue Y Xu J . Dysbiosis signatures of the microbial profile in tissue from bladder cancer. Cancer Med. (2019) 8:6904–14. doi: 10.1002/cam4.2419, PMID: 31568654 PMC6854010

[42] Mansour B Monyók Á Makra N Gajdács M Vadnay I Ligeti B . Bladder cancer-related microbiota: examining differences in urine and tissue samples. Sci Rep. (2020) 10:11042. doi: 10.1038/s41598-020-67443-2, PMID: 32632181 PMC7338485

[43] Parra-Grande M Oré-Arce M Martínez-Priego L D’Auria G Rosselló-Mora R Lillo M . Profiling the bladder microbiota in patients with bladder cancer. Front Microbiol. (2021) 12718776:718776. doi: 10.3389/fmicb.2021.718776, PMID: 35197936 PMC8859159

[44] Pederzoli F Ferrarese R Amato V Locatelli I Alchera E Lucianò R . Sex-specific alterations in the urinary and tissue microbiome in therapy-naïve urothelial bladder cancer patients. Eur Urol Oncol. (2020) 3:784–8. doi: 10.1016/j.euo.2020.04.002, PMID: 32345542

[45] Yao R Ai B Wang Z Shen B Xue G Yu D . Uncovering microbial composition of the tissue microenvironment in bladder cancer using RNA sequencing data. J Cancer. (2024) 15:2431–41. doi: 10.7150/jca.93055, PMID: 38495492 PMC10937280

[46] Oresta B Braga D Lazzeri M Frego N Saita A Faccani C . The microbiome of catheter collected urine in males with bladder cancer according to disease stage. J Urol. (2021) 205:86–93. doi: 10.1097/ju.0000000000001336, PMID: 32856979

[47] Sun JX Xia QD Zhong XY Liu Z Wang SG . The bladder microbiome of NMIBC and MIBC patients revealed by 2bRAD-M. Front Cell Infect Microbiol. (2023) 131182322:1182322. doi: 10.3389/fcimb.2023.1182322, PMID: 37351184 PMC10282653

[48] Bilski K Żeber-Lubecka N Kulecka M Dąbrowska M Bałabas A Ostrowski J . Microbiome sex-related diversity in non-muscle-invasive urothelial bladder cancer. Curr Issues Mol Biol. (2024) 46:3595–609. doi: 10.3390/cimb46040225, PMID: 38666955 PMC11048804

[49] Knorr J Lone Z Werneburg G Adler A Agudelo J Suryavanshi M . An exploratory study investigating the impact of the bladder tumor microbiome on Bacillus Calmette Guerin (BCG) response in non-muscle invasive bladder cancer. Urol Oncol. (2024) 42:291.e291–291.e211. doi: 10.1016/j.urolonc.2024.04.011, PMID: 38664180

[50] Hussein AA Bhat TA Jing Z Gomez EC Wasay MA Singh PK . Does the urinary microbiome profile change after treatment of bladder cancer? World J Urol. (2023) 41:3593–8. doi: 10.1007/s00345-023-04627-1, PMID: 37796319

[51] Qiu Y Gao Y Chen C Xie M Huang P Sun Q . Deciphering the influence of urinary microbiota on FoxP3+ regulatory T cell infiltration and prognosis in Chinese patients with non-muscle-invasive bladder cancer. Hum Cell. (2022) 35:511–21. doi: 10.1007/s13577-021-00659-0, PMID: 35032011

[52] Kamat AM Sylvester RJ Böhle A Palou J Lamm DL Brausi M . Definitions, end points, and clinical trial designs for non-muscle-invasive bladder cancer: recommendations from the international bladder cancer group. J Clin Oncol. (2016) 34:1935–44. doi: 10.1200/jco.2015.64.4070, PMID: 26811532 PMC5321095

[53] Chang SS Bochner BH Chou R Dreicer R Kamat AM Lerner SP . Treatment of non-metastatic muscle-invasive bladder cancer: AUA/ASCO/ASTRO/SUO guideline. J Urol. (2017) 198:552–9. doi: 10.1016/j.juro.2017.04.086, PMID: 28456635 PMC5626446

[54] Ghodoussipour S Bivalacqua T Bryan RT Li R Mir MC Palou J . A systematic review of novel intravesical approaches for the treatment of patients with non-muscle-invasive bladder cancer. Eur Urol. (2025) 88:33–55. doi: 10.1016/j.eururo.2025.02.010, PMID: 40253283

[55] Heidrich V Mariotti ACH Inoue LT Coser EM Dos Santos EX Dos Santos HDB . The bladder microbiota is not significantly altered by intravesical BCG therapy. Urol Oncol. (2024) 42:22.e13–21. doi: 10.1016/j.urolonc.2023.11.003, PMID: 38030469

[56] Min K Zheng CM Kim S Kim H Lee M Piao XM . Differential urinary microbiome and its metabolic footprint in bladder cancer patients following BCG treatment. Int J Mol Sci. (2024) 25:11157. doi: 10.3390/ijms252011157, PMID: 39456941 PMC11508893

[57] Isali I Almassi N Nizam A Campbell R Weight C Gupta S . State of the art: the microbiome in bladder cancer. Urol Oncol. (2025) 43:199–208. doi: 10.1016/j.urolonc.2024.11.008, PMID: 39581825

[58] Zhang Y Wang W Zhou H Cui Y . Urinary Eubacterium sp. CAG:581 Promotes Non-Muscle Invasive Bladder Cancer (NMIBC) Development through the ECM1/MMP9 Pathway. Cancers (Basel). (2023) 15:809. doi: 10.3390/cancers15030809, PMID: 36765767 PMC9913387

[59] Camargo JA Passos GR Ferrari KL Billis A Saad MJA Reis LO . Intravesical immunomodulatory imiquimod enhances bacillus calmette-guérin downregulation of nonmuscle-invasive bladder cancer. Clin Genitourin Cancer. (2018) 16:e587–93. doi: 10.1016/j.clgc.2017.10.019, PMID: 29174504

[60] He C Li B Huang L Teng C Bao Y Ren M . Gut microbial composition changes in bladder cancer patients: A case-control study in Harbin, China. Asia Pac J Clin Nutr. (2020) 29:395–403. doi: 10.6133/apjcn.202007_29(2).0022, PMID: 32674247

[61] Yang H Jin C Li J Zhang Z Zhao K Yin X . Causal relationship between bladder cancer and gut microbiota contributes to the gut-bladder axis: A two-sample Mendelian randomization study. Urol Oncol. (2024) 43:267. doi: 10.1016/j.urolonc.2024.10.014, PMID: 39489648

[62] Mingdong W Xiang G Yongjun Q Mingshuai W Hao P . Causal associations between gut microbiota and urological tumors: a two-sample mendelian randomization study. BMC Cancer. (2023) 23:854. doi: 10.1186/s12885-023-11383-3, PMID: 37697271 PMC10496293

[63] Wang B Qiu Y Xie M Huang P Yu Y Sun Q . Gut microbiota Parabacteroides distasonis enchances the efficacy of immunotherapy for bladder cancer by activating anti-tumor immune responses. BMC Microbiol. (2024) 24:237. doi: 10.1186/s12866-024-03372-8, PMID: 38961326 PMC11221038

[64] Li WT Iyangar AS Reddy R Chakladar J Bhargava V Sakamoto K . The bladder microbiome is associated with epithelial-mesenchymal transition in muscle invasive urothelial bladder carcinoma. Cancers (Basel). (2021) 13:3649. doi: 10.3390/cancers13153649, PMID: 34359550 PMC8344975

[65] Galeano Niño JL Wu H LaCourse KD Kempchinsky AG Baryiames A Barber B . Effect of the intratumoral microbiota on spatial and cellular heterogeneity in cancer. Nature. (2022) 611:810–7. doi: 10.1038/s41586-022-05435-0, PMID: 36385528 PMC9684076

[66] Woolley DE Grafton CA . Collagenase immunolocalization studies of cutaneous secondary melanomas. Br J Cancer. (1980) 42:260–5. doi: 10.1038/bjc.1980.225, PMID: 6252926 PMC2010383

[67] de Almeida LGN Thode H Eslambolchi Y Chopra S Young D Gill S . Matrix metalloproteinases: from molecular mechanisms to physiology, pathophysiology, and pharmacology. Pharmacol Rev. (2022) 74:712–68. doi: 10.1124/pharmrev.121.000349, PMID: 35738680

[68] Wysocki AB Bhalla-Regev SK Tierno PM Jr. Stevens-Riley M Wiygul RC . Proteolytic activity by multiple bacterial species isolated from chronic venous leg ulcers degrades matrix substrates. Biol Res Nurs. (2013) 15:407–15. doi: 10.1177/1099800412464683, PMID: 23118301

[69] Langston JP Carson CC 3rd . Peyronie’s disease: review and recent advances. Maturitas. (2014) 78:341–3. doi: 10.1016/j.maturitas.2014.05.024, PMID: 24984940

[70] Watanabe K . Collagenolytic proteases from bacteria. Appl Microbiol Biotechnol. (2004) 63:520–6. doi: 10.1007/s00253-003-1442-0, PMID: 14556041

[71] Goekeri C Linke KAK Hoffmann K Lopez-Rodriguez E Gluhovic V Voß A . Enzymatic modulation of the pulmonary glycocalyx enhances susceptibility to streptococcus pneumoniae. Am J Respir Cell Mol Biol. (2024) 71:646–58. doi: 10.1165/rcmb.2024-0003OC, PMID: 39042016 PMC11622634

[72] Theander TG Kharazmi A Pedersen BK Christensen LD Tvede N Poulsen LK . Inhibition of human lymphocyte proliferation and cleavage of interleukin-2 by Pseudomonas aeruginosa proteases. Infect Immun. (1988) 56:1673–7. doi: 10.1128/iai.56.7.1673-1677.1988, PMID: 3133317 PMC259461

[73] Wilson M Seymour R Henderson B . Bacterial perturbation of cytokine networks. Infect Immun. (1998) 66:2401–9. doi: 10.1128/iai.66.6.2401-2409.1998, PMID: 9596695 PMC108217

[74] Maresz KJ Hellvard A Sroka A Adamowicz K Bielecka E Koziel J . Porphyromonas gingivalis facilitates the development and progression of destructive arthritis through its unique bacterial peptidylarginine deiminase (PAD). PloS Pathog. (2013) 9:e1003627. doi: 10.1371/journal.ppat.1003627, PMID: 24068934 PMC3771902

[75] Zeltz C Gullberg D . Post-translational modifications of integrin ligands as pathogenic mechanisms in disease. Matrix Biol. (2014) 40:5–9. doi: 10.1016/j.matbio.2014.08.001, PMID: 25116951

[76] Xu X Wei F Xiao L Wu R Wei B Huang S . High proportion of circulating CD8 + CD28- senescent T cells is an independent predictor of distant metastasis in nasopharyngeal canrcinoma after radiotherapy. J Transl Med. (2023) 21:64. doi: 10.1186/s12967-023-03912-2, PMID: 36721233 PMC9887944

[77] Mace EM Orange JS . Emerging insights into human health and NK cell biology from the study of NK cell deficiencies. Immunol Rev. (2019) 287:202–25. doi: 10.1111/imr.12725, PMID: 30565241 PMC6310041

[78] Kakuda T Suzuki J Matsuoka Y Kikugawa T Saika T Yamashita M . Senescent CD8(+) T cells acquire NK cell-like innate functions to promote antitumor immunity. Cancer Sci. (2023) 114:2810–20. doi: 10.1111/cas.15824, PMID: 37186472 PMC10323091

[79] Shin E Bak SH Park T Kim JW Yoon SR Jung H . Understanding NK cell biology for harnessing NK cell therapies: targeting cancer and beyond. Front Immunol. (2023) 141192907:1192907. doi: 10.3389/fimmu.2023.1192907, PMID: 37539051 PMC10395517

[80] Nuñez C Nishimoto N Gartland GL Billips LG Burrows PD Kubagawa H . B cells are generated throughout life in humans. J Immunol. (1996) 156:866–72. doi: 10.4049/jimmunol.156.2.866, PMID: 8543844

[81] de Mol J Kuiper J Tsiantoulas D Foks AC . The dynamics of B cell aging in health and disease. Front Immunol. (2021) 12733566:733566. doi: 10.3389/fimmu.2021.733566, PMID: 34675924 PMC8524000

[82] Cakala-Jakimowicz M Kolodziej-Wojnar P Puzianowska-Kuznicka M . Aging-related cellular, structural and functional changes in the lymph nodes: A significant component of immunosenescence? An overview. Cells. (2021) 10:3148. doi: 10.3390/cells10113148, PMID: 34831371 PMC8621398

[83] Turner VM Mabbott NA . Structural and functional changes to lymph nodes in ageing mice. Immunology. (2017) 151:239–47. doi: 10.1111/imm.12727, PMID: 28207940 PMC5418465

[84] Lanfermeijer J Borghans JAM van Baarle D . How age and infection history shape the antigen-specific CD8(+) T-cell repertoire: Implications for vaccination strategies in older adults. Aging Cell. (2020) 19:e13262. doi: 10.1111/acel.13262, PMID: 33078890 PMC7681067

[85] Miller J . The function of the thymus and its impact on modern medicine. Science. (2020) 369:eaba2429. doi: 10.1126/science.aba2429, PMID: 32732394

[86] Wilkinson AC Igarashi KJ Nakauchi H . Haematopoietic stem cell self-renewal *in vivo* and ex vivo. Nat Rev Genet. (2020) 21:541–54. doi: 10.1038/s41576-020-0241-0, PMID: 32467607 PMC7894993

[87] Frasca D Diaz A Romero M Mendez NV Landin AM Blomberg BB . Effects of age on H1N1-specific serum IgG1 and IgG3 levels evaluated during the 2011–2012 influenza vaccine season. Immun Ageing. (2013) 10:14. doi: 10.1186/1742-4933-10-14, PMID: 23607926 PMC3639840

[88] Dowery R Benhamou D Benchetrit E Harel O Nevelsky A Zisman-Rozen S . Peripheral B cells repress B-cell regeneration in aging through a TNF-α/IGFBP-1/IGF-1 immune-endocrine axis. Blood. (2021) 138:1817–29. doi: 10.1182/blood.2021012428, PMID: 34297797 PMC9642783

[89] Li J Huang D Lei B Huang J Yang L Nie M . VLA-4 suppression by senescence signals regulates meningeal immunity and leptomeningeal metastasis. Elife. (2022) 11:e83272. doi: 10.7554/eLife.83272, PMID: 36484779 PMC9803356

[90] Liu X Li L Si F Huang L Zhao Y Zhang C . NK and NKT cells have distinct properties and functions in cancer. Oncogene. (2021) 40:4521–37. doi: 10.1038/s41388-021-01880-9, PMID: 34120141 PMC12416827

[91] Manser AR Uhrberg M . Age-related changes in natural killer cell repertoires: impact on NK cell function and immune surveillance. Cancer Immunol Immunother. (2016) 65:417–26. doi: 10.1007/s00262-015-1750-0, PMID: 26288343 PMC11028690

[92] Shehata HM Hoebe K Chougnet CA . The aged nonhematopoietic environment impairs natural killer cell maturation and function. Aging Cell. (2015) 14:191–9. doi: 10.1111/acel.12303, PMID: 25677698 PMC4364831

[93] Agrawal A Gupta S . Impact of aging on dendritic cell functions in humans. Ageing Res Rev. (2011) 10:336–45. doi: 10.1016/j.arr.2010.06.004, PMID: 20619360 PMC3030666

[94] Gardner JK Mamotte CDS Jackaman C Nelson DJ . Modulation of dendritic cell and T cell cross-talk during aging: The potential role of checkpoint inhibitory molecules. Ageing Res Rev. (2017) 38:40–51. doi: 10.1016/j.arr.2017.07.002, PMID: 28736117

[95] Gon Y Hashimoto S Hayashi S Koura T Matsumoto K Horie T . Lower serum concentrations of cytokines in elderly patients with pneumonia and the impaired production of cytokines by peripheral blood monocytes in the elderly. Clin Exp Immunol. (1996) 106:120–6., PMID: 8870709

[96] van Duin D Allore HG Mohanty S Ginter S Newman FK Belshe RB . Prevaccine determination of the expression of costimulatory B7 molecules in activated monocytes predicts influenza vaccine responses in young and older adults. J Infect Dis. (2007) 195:1590–7. doi: 10.1086/516788, PMID: 17471428

[97] Liang S Domon H Hosur KB Wang M Hajishengallis G . Age-related alterations in innate immune receptor expression and ability of macrophages to respond to pathogen challenge. vitro. Mech Ageing Dev. (2009) 130:538–46. doi: 10.1016/j.mad.2009.06.006, PMID: 19559723 PMC2717634

[98] Jackaman C Radley-Crabb HG Soffe Z Shavlakadze T Grounds MD Nelson DJ . Targeting macrophages rescues age-related immune deficiencies in C57BL/6J geriatric mice. Aging Cell. (2013) 12:345–57. doi: 10.1111/acel.12062, PMID: 23442123

[99] Sharma S Dominguez AL Lustgarten J . High accumulation of T regulatory cells prevents the activation of immune responses in aged animals. J Immunol. (2006) 177:8348–55. doi: 10.4049/jimmunol.177.12.8348, PMID: 17142731

[100] Fontana L Zhao E Amir M Dong H Tanaka K Czaja MJ . Aging promotes the development of diet-induced murine steatohepatitis but not steatosis. Hepatology. (2013) 57:995–1004. doi: 10.1002/hep.26099, PMID: 23081825 PMC3566282

[101] Kelly J Ali Khan A Yin J Ferguson TA Apte RS . Senescence regulates macrophage activation and angiogenic fate at sites of tissue injury in mice. J Clin Invest. (2007) 117:3421–6. doi: 10.1172/jci32430, PMID: 17975672 PMC2045608

[102] Franceschi C Garagnani P Parini P Giuliani C Santoro A . Inflammaging: a new immune-metabolic viewpoint for age-related diseases. Nat Rev Endocrinol. (2018) 14:576–90. doi: 10.1038/s41574-018-0059-4, PMID: 30046148

[103] Thevaranjan N Puchta A Schulz C Naidoo A Szamosi JC Verschoor CP . Age-associated microbial dysbiosis promotes intestinal permeability, systemic inflammation, and macrophage dysfunction. Cell Host Microbe. (2017) 21:455–466.e454. doi: 10.1016/j.chom.2017.03.002, PMID: 28407483 PMC5392495

[104] Martínez-López MF de Almeida CR Fontes M Mendes RV Kaufmann SHE Fior R . Macrophages directly kill bladder cancer cells through TNF signaling as an early response to BCG therapy. Dis Model Mech. (2024) 17:dmm050693. doi: 10.1242/dmm.050693, PMID: 39114912 PMC11554267

[105] Tan C Li C Ge R Zhang W Wu Z Wang S . Mcl-1 downregulation enhances BCG treatment efficacy in bladder cancer by promoting macrophage polarization. Cancer Cell Int. (2025) 25:48. doi: 10.1186/s12935-025-03676-3, PMID: 39955585 PMC11830210

[106] Xu J Ding L Mei J Hu Y Kong X Dai S . Dual roles and therapeutic targeting of tumor-associated macrophages in tumor microenvironments. Signal Transduct Target Ther. (2025) 10:268. doi: 10.1038/s41392-025-02325-5, PMID: 40850976 PMC12375796

[107] Duan Z Luo Y . Targeting macrophages in cancer immunotherapy. Signal Transduct Target Ther. (2021) 6:127. doi: 10.1038/s41392-021-00506-6, PMID: 33767177 PMC7994399

[108] Jian N Yu L Ma L Zheng B Huang W . BCG therapy in bladder cancer and its tumor microenvironment interactions. Clin Microbiol Rev. (2025) 38:e0021224. doi: 10.1128/cmr.00212-24, PMID: 40111053 PMC12180517

[109] Salminen A Kauppinen A Kaarniranta K . Myeloid-derived suppressor cells (MDSC): an important partner in cellular/tissue senescence. Biogerontology. (2018) 19:325–39. doi: 10.1007/s10522-018-9762-8, PMID: 29959657

[110] Salminen A Kauppinen A Kaarniranta K . AMPK activation inhibits the functions of myeloid-derived suppressor cells (MDSC): impact on cancer and aging. J Mol Med (Berl). (2019) 97:1049–64. doi: 10.1007/s00109-019-01795-9, PMID: 31129755 PMC6647228

[111] Ecker BL Kaur A Douglass SM Webster MR Almeida FV Marino GE . Age-related changes in HAPLN1 increase lymphatic permeability and affect routes of melanoma metastasis. Cancer Discov. (2019) 9:82–95. doi: 10.1158/2159-8290.Cd-18-0168, PMID: 30279172 PMC6328344

[112] Kaur A Webster MR Marchbank K Behera R Ndoye A Kugel CH 3rd . sFRP2 in the aged microenvironment drives melanoma metastasis and therapy resistance. Nature. (2016) 532:250–4. doi: 10.1038/nature17392, PMID: 27042933 PMC4833579

[113] Bancaro N Calì B Troiani M Elia AR Arzola RA Attanasio G . Apolipoprotein E induces pathogenic senescent-like myeloid cells in prostate cancer. Cancer Cell. (2023) 41:602–619.e611. doi: 10.1016/j.ccell.2023.02.004, PMID: 36868226

[114] Choi UY Choi YJ Lee SA Yoo JS . Cisd2 deficiency impairs neutrophil function by regulating calcium homeostasis via Calnexin and SERCA. BMB Rep. (2024) 57:256–61. doi: 10.5483/BMBRep.2024-0011, PMID: 38627949 PMC11139677

[115] Yang C Wang Z Li L Zhang Z Jin X Wu P . Aged neutrophils form mitochondria-dependent vital NETs to promote breast cancer lung metastasis. J Immunother Cancer. (2021) 9:e002875. doi: 10.1136/jitc-2021-002875, PMID: 34716206 PMC8559246

[116] Si F Liu X Tao Y Zhang Y Ma F Hsueh EC . Blocking senescence and tolerogenic function of dendritic cells induced by γδ Treg cells enhances tumor-specific immunity for cancer immunotherapy. J Immunother Cancer. (2024) 12:e008219. doi: 10.1136/jitc-2023-008219, PMID: 38580332 PMC11002396

[117] Piskor EM Ross J Möröy T Kosan C . Myc-interacting zinc finger protein 1 (Miz-1) is essential to maintain homeostasis and immunocompetence of the B cell lineage. Biol (Basel). (2022) 11:504. doi: 10.3390/biology11040504, PMID: 35453704 PMC9027237

[118] Frasca D Romero M Garcia D Thaller S Bueno V . Adipocyte-derived inflammatory molecules induce senescent B cells through metabolic pathways. Obes (Silver Spring). (2024) 32:1441–7. doi: 10.1002/oby.24013, PMID: 38575197 PMC11269042

[119] Khan S Chakraborty M Wu F Chen N Wang T Chan YT . B cells promote T cell immunosenescence and mammalian aging parameters. bioRxiv. (2023) 556363. doi: 10.1101/2023.09.12.556363, PMID: 38529494 PMC10962733

[120] Wang SS Liu W Ly D Xu H Qu L Zhang L . Tumor-infiltrating B cells: their role and application in anti-tumor immunity in lung cancer. Cell Mol Immunol. (2019) 16:6–18. doi: 10.1038/s41423-018-0027-x, PMID: 29628498 PMC6318290

[121] Kogut I Scholz JL Cancro MP Cambier JC . B cell maintenance and function in aging. Semin Immunol. (2012) 24:342–9. doi: 10.1016/j.smim.2012.04.004, PMID: 22560930

[122] Medzhitov R . Toll-like receptors and innate immunity. Nat Rev Immunol. (2001) 1:135–45. doi: 10.1038/35100529, PMID: 11905821

[123] Karin M Ben-Neriah Y . Phosphorylation meets ubiquitination: the control of NF-[kappa]B activity. Annu Rev Immunol. (2000) 18:621–63. doi: 10.1146/annurev.immunol.18.1.621, PMID: 10837071

[124] Zarember KA Godowski PJ . Tissue expression of human Toll-like receptors and differential regulation of Toll-like receptor mRNAs in leukocytes in response to microbes, their products, and cytokines. J Immunol. (2002) 168:554–61. doi: 10.4049/jimmunol.168.2.554, PMID: 11777946

[125] Baggiolini M Dewald B Moser B . Human chemokines: an update. Annu Rev Immunol. (1997) 15:675–705. doi: 10.1146/annurev.immunol.15.1.675, PMID: 9143704

[126] Amulic B Cazalet C Hayes GL Metzler KD Zychlinsky A . Neutrophil function: from mechanisms to disease. Annu Rev Immunol. (2012) 30:459–89. doi: 10.1146/annurev-immunol-020711-074942, PMID: 22224774

[127] Brinkmann V Reichard U Goosmann C Fauler B Uhlemann Y Weiss DS . Neutrophil extracellular traps kill bacteria. Science. (2004) 303:1532–5. doi: 10.1126/science.1092385, PMID: 15001782

[128] Nathan C . Neutrophils and immunity: challenges and opportunities. Nat Rev Immunol. (2006) 6:173–82. doi: 10.1038/nri1785, PMID: 16498448

[129] Nathan C . Points of control in inflammation. Nature. (2002) 420:846–52. doi: 10.1038/nature01320, PMID: 12490957

[130] Sharma P Vijaykumar A Raghavan JV Rananaware SR Alakesh A Bodele J . Particle uptake driven phagocytosis in macrophages and neutrophils enhances bacterial clearance. J Contr Rel. (2022) 343:131–41. doi: 10.1016/j.jconrel.2022.01.030, PMID: 35085696 PMC7615985

[131] Aderem A Underhill DM . Mechanisms of phagocytosis in macrophages. Annu Rev Immunol. (1999) 17:593–623. doi: 10.1146/annurev.immunol.17.1.593, PMID: 10358769

[132] Banchereau J Steinman RM . Dendritic cells and the control of immunity. Nature. (1998) 392:245–52. doi: 10.1038/32588, PMID: 9521319

[133] Zhu J Paul WE . CD4 T cells: fates, functions, and faults. Blood. (2008) 112:1557–69. doi: 10.1182/blood-2008-05-078154, PMID: 18725574 PMC2518872

[134] Kaech SM Cui W . Transcriptional control of effector and memory CD8+ T cell differentiation. Nat Rev Immunol. (2012) 12:749–61. doi: 10.1038/nri3307, PMID: 23080391 PMC4137483

[135] Butler DSC Cafaro C Putze J Wan MLY Tran TH Ambite I . A bacterial protease depletes c-MYC and increases survival in mouse models of bladder and colon cancer. Nat Biotechnol. (2021) 39:754–64. doi: 10.1038/s41587-020-00805-3, PMID: 33574609

[136] Mehmandar-Oskuie A Tohidfar M Hajikhani B Karimi F . Anticancer effects of cell-free culture supernatant of Escherichia coli in bladder cancer cell line: New insight into the regulation of inflammation. Gene. (2023) 889:147795. doi: 10.1016/j.gene.2023.147795, PMID: 37708921

[137] Andersson M Poljakovic M Persson K . Caspase-3-dependent apoptosis in Escherichia coli-infected urothelium: involvement of inducible nitric oxide synthase. BJU Int. (2006) 98:160–5. doi: 10.1111/j.1464-410X.2006.06151.x, PMID: 16831162

[138] Abraham SN Miao Y . The nature of immune responses to urinary tract infections. Nat Rev Immunol. (2015) 15:655–63. doi: 10.1038/nri3887, PMID: 26388331 PMC4926313

[139] Murray BO Flores C Williams C Flusberg DA Marr EE Kwiatkowska KM . Recurrent urinary tract infection: A mystery in search of better model systems. Front Cell Infect Microbiol. (2021) 11691210:691210. doi: 10.3389/fcimb.2021.691210, PMID: 34123879 PMC8188986

[140] Mulvey MA Lopez-Boado YS Wilson CL Roth R Parks WC Heuser J . Induction and evasion of host defenses by type 1-piliated uropathogenic Escherichia coli. Science. (1998) 282:1494–7. doi: 10.1126/science.282.5393.1494, PMID: 9822381

[141] Justice SS Hung C Theriot JA Fletcher DA Anderson GG Footer MJ . Differentiation and developmental pathways of uropathogenic Escherichia coli in urinary tract pathogenesis. Proc Natl Acad Sci U.S.A. (2004) 101:1333–8. doi: 10.1073/pnas.0308125100, PMID: 14739341 PMC337053

[142] Wright KJ Seed PC Hultgren SJ . Uropathogenic Escherichia coli flagella aid in efficient urinary tract colonization. Infect Immun. (2005) 73:7657–68. doi: 10.1128/iai.73.11.7657-7668.2005, PMID: 16239570 PMC1273872

[143] Martinez JJ Mulvey MA Schilling JD Pinkner JS Hultgren SJ . Type 1 pilus-mediated bacterial invasion of bladder epithelial cells. EMBO J. (2000) 19:2803–12. doi: 10.1093/emboj/19.12.2803, PMID: 10856226 PMC203355

[144] Choi HW Bowen SE Miao Y Chan CY Miao EA Abrink M . Loss of bladder epithelium induced by cytolytic mast cell granules. Immunity. (2016) 45:1258–69. doi: 10.1016/j.immuni.2016.11.003, PMID: 27939674 PMC5177478

[145] Chen MC Blunt LW Pins MR Klumpp DJ . Tumor necrosis factor promotes differential trafficking of bladder mast cells in neurogenic cystitis. J Urol. (2006) 175:754–9. doi: 10.1016/s0022-5347(05)00171-0, PMID: 16407045

[146] Chen MC Keshavan P Gregory GD Klumpp DJ . RANTES mediates TNF-dependent lamina propria mast cell accumulation and barrier dysfunction in neurogenic cystitis. Am J Physiol Renal Physiol. (2007) 292:F1372–1379. doi: 10.1152/ajprenal.00472.2006, PMID: 17244892

[147] Wernersson S Pejler G . Mast cell secretory granules: armed for battle. Nat Rev Immunol. (2014) 14:478–94. doi: 10.1038/nri3690, PMID: 24903914

[148] Nagamatsu K Hannan TJ Guest RL Kostakioti M Hadjifrangiskou M Binkley J . Dysregulation of Escherichia coli α-hemolysin expression alters the course of acute and persistent urinary tract infection. Proc Natl Acad Sci U.S.A. (2015) 112:E871–880. doi: 10.1073/pnas.1500374112, PMID: 25675528 PMC4345586

[149] Broz P von Moltke J Jones JW Vance RE Monack DM . Differential requirement for Caspase-1 autoproteolysis in pathogen-induced cell death and cytokine processing. Cell Host Microbe. (2010) 8:471–83. doi: 10.1016/j.chom.2010.11.007, PMID: 21147462 PMC3016200

[150] Haynes MD Martin TA Jenkins SA Kynaston HG Matthews PN Jiang WG . Tight junctions and bladder cancer (review). Int J Mol Med. (2005) 16:3–9. doi: 10.3892/ijmm.16.1.3, PMID: 15942671

[151] Otani T Furuse M . Tight junction structure and function revisited. Trends Cell Biol. (2020) 30:805–17. doi: 10.1016/j.tcb.2020.08.004, PMID: 32891490

[152] Tsukita S Furuse M . Occludin and claudins in tight-junction strands: leading or supporting players? Trends Cell Biol. (1999) 9:268–73. doi: 10.1016/s0962-8924(99)01578-0, PMID: 10370242

[153] Tsukita S Tanaka H Tamura A . The claudins: from tight junctions to biological systems. Trends Biochem Sci. (2019) 44:141–52. doi: 10.1016/j.tibs.2018.09.008, PMID: 30665499

[154] Montalbetti N Rued AC Taiclet SN Birder LA Kullmann FA Carattino MD . Urothelial tight junction barrier dysfunction sensitizes bladder afferents. eNeuro. (2017) 4:ENEURO.0381-16.2017. doi: 10.1523/eneuro.0381-16.2017, PMID: 28560313 PMC5442440

[155] Zhang CO Wang JY Koch KR Keay S . Regulation of tight junction proteins and bladder epithelial paracellular permeability by an antiproliferative factor from patients with interstitial cystitis. J Urol. (2005) 174:2382–7. doi: 10.1097/01.ju.0000180417.11976.99, PMID: 16280852

[156] Gao J Young G Xue KX Li BG Sun YL . Characteristics of invasiveness of human nasopharyngeal carcinoma cells in organ culture, as observed by scanning electron microscopy. Pathol Res Pract. (1982) 174:325–41. doi: 10.1016/S0344-0338(82)80015-0, PMID: 7155978

[157] Pinho SS Reis CA . Glycosylation in cancer: mechanisms and clinical implications. Nat Rev Cancer. (2015) 15:540–55. doi: 10.1038/nrc3982, PMID: 26289314

[158] Alfano M Canducci F Nebuloni M Clementi M Montorsi F Salonia A . The interplay of extracellular matrix and microbiome in urothelial bladder cancer. Nat Rev Urol. (2016) 13:77–90. doi: 10.1038/nrurol.2015.292, PMID: 26666363 PMC7097604

[159] Ganz T . Defensins: antimicrobial peptides of innate immunity. Nat Rev Immunol. (2003) 3:710–20. doi: 10.1038/nri1180, PMID: 12949495

[160] Zasloff M . Antimicrobial peptides, innate immunity, and the normally sterile urinary tract. J Am Soc Nephrol. (2007) 18:2810–6. doi: 10.1681/asn.2007050611, PMID: 17942949

[161] Sobel JD . Pathogenesis of urinary tract infection. Role Host defenses. Infect Dis Clin North Am. (1997) 11:531–49. doi: 10.1016/s0891-5520(05)70372-x, PMID: 9378922

[162] Billips BK Forrestal SG Rycyk MT Johnson JR Klumpp DJ Schaeffer AJ . Modulation of host innate immune response in the bladder by uropathogenic Escherichia coli. Infect Immun. (2007) 75:5353–60. doi: 10.1128/iai.00922-07, PMID: 17724068 PMC2168307

[163] Blobe GC Schiemann WP Lodish HF . Role of transforming growth factor beta in human disease. N Engl J Med. (2000) 342:1350–8. doi: 10.1056/nejm200005043421807, PMID: 10793168

[164] Moore KW de Waal Malefyt R Coffman RL O’Garra A . Interleukin-10 and the interleukin-10 receptor. Annu Rev Immunol. (2001) 19:683–765. doi: 10.1146/annurev.immunol.19.1.683, PMID: 11244051

[165] Xu Y Zeng H Jin K Liu Z Zhu Y Xu L . Immunosuppressive tumor-associated macrophages expressing interlukin-10 conferred poor prognosis and therapeutic vulnerability in patients with muscle-invasive bladder cancer. J Immunother Cancer. (2022) 10:e003416. doi: 10.1136/jitc-2021-003416, PMID: 35338085 PMC8961180

[166] Chen Z Zhou L Liu L Hou Y Xiong M Yang Y . Single-cell RNA sequencing highlights the role of inflammatory cancer-associated fibroblasts in bladder urothelial carcinoma. Nat Commun. (2020) 11:5077. doi: 10.1038/s41467-020-18916-5, PMID: 33033240 PMC7545162

[167] Whiteside TL . Regulatory T cell subsets in human cancer: are they regulating for or against tumor progression? Cancer Immunol Immunother. (2014) 63:67–72. doi: 10.1007/s00262-013-1490-y, PMID: 24213679 PMC3888225

[168] Ritz U Seliger B . The transporter associated with antigen processing (TAP): structural integrity, expression, function, and its clinical relevance. Mol Med. (2001) 7:149–58. doi: 10.1007/BF03401948, PMID: 11471551 PMC1950029

[169] Zheng X Chen J Deng M Ning K Peng Y Liu Z . G3BP1 and SLU7 jointly promote immune evasion by downregulating MHC-I via PI3K/akt activation in bladder cancer. Adv Sci (Weinh). (2024) 11:e2305922. doi: 10.1002/advs.202305922, PMID: 38084438 PMC10870071

[170] DeJong EN Surette MG Bowdish DME . The gut microbiota and unhealthy aging: disentangling cause from consequence. Cell Host Microbe. (2020) 28:180–9. doi: 10.1016/j.chom.2020.07.013, PMID: 32791111

[171] Madison AA Burd CE Andridge R Wilson SJ Bailey MT Belury MA . Gut microbiota richness and diversity track with T cell aging in healthy adults. J Gerontol A Biol Sci Med Sci. (2024) 79:glad276. doi: 10.1093/gerona/glad276, PMID: 38123141 PMC10878250

[172] Chulenbayeva L Ganzhula Y Kozhakhmetov S Jarmukhanov Z Nurgaziyev M Nurgozhina A . The trajectory of successful aging: insights from metagenome and cytokine profiling. Gerontology. (2024) 70:390–407. doi: 10.1159/000536082, PMID: 38246133 PMC11008724

[173] Kawamoto S Maruya M Kato LM Suda W Atarashi K Doi Y . Foxp3(+) T cells regulate immunoglobulin a selection and facilitate diversification of bacterial species responsible for immune homeostasis. Immunity. (2014) 41:152–65. doi: 10.1016/j.immuni.2014.05.016, PMID: 25017466

[174] Bashir H Singh S Singh RP Agrewala JN Kumar R . Age-mediated gut microbiota dysbiosis promotes the loss of dendritic cells tolerance. Aging Cell. (2023) 22:e13838. doi: 10.1111/acel.13838, PMID: 37161603 PMC10265174

[175] Sbierski-Kind J Grenkowitz S Schlickeiser S Sandforth A Friedrich M Kunkel D . Effects of caloric restriction on the gut microbiome are linked with immune senescence. Microbiome. (2022) 10:57. doi: 10.1186/s40168-022-01249-4, PMID: 35379337 PMC8978410

[176] Zhu X Huang X Hu M Sun R Li J Wang H . A specific enterotype derived from gut microbiome of older individuals enables favorable responses to immune checkpoint blockade therapy. Cell Host Microbe. (2024) 32:489–505.e485. doi: 10.1016/j.chom.2024.03.002, PMID: 38513657

[177] Gopalakrishnan V Helmink BA Spencer CN Reuben A Wargo JA . The influence of the gut microbiome on cancer, immunity, and cancer immunotherapy. Cancer Cell. (2018) 33:570–80. doi: 10.1016/j.ccell.2018.03.015, PMID: 29634945 PMC6529202

[178] Trầ;n TA Lee HY Choi HW . Metabolite-mediated mechanisms linking the urinary microbiome to bladder cancer. J Microbiol. (2025) 63:e2509001. doi: 10.71150/jm.2509001, PMID: 41309232 PMC13577029

[179] Bender MJ McPherson AC Phelps CM Pandey SP Laughlin CR Shapira JH . Dietary tryptophan metabolite released by intratumoral Lactobacillus reuteri facilitates immune checkpoint inhibitor treatment. Cell. (2023) 186:1846–1862.e1826. doi: 10.1016/j.cell.2023.03.011, PMID: 37028428 PMC10148916

[180] Yue L Geng F Jin J Li W Liu B Du M . Lactobacillus reuteri assists engineered bacteria that target tumors to release PD-L1nb to mitigate the adverse effects of breast cancer immunotherapy. Biotechnol J. (2024) 19:e202400428. doi: 10.1002/biot.202400428, PMID: 39711089

[181] Soliman A Murad MR Jabrieh G AlEdani EM Saeed A Belabaci Z . A systematic review and meta-analysis of the effectiveness and safety of immune checkpoint inhibitors in patients with BCG-unresponsive non-muscle-invasive bladder cancer. Clin Genitourin Cancer. (2025) 23:102445. doi: 10.1016/j.clgc.2025.102445, PMID: 41139553

[182] Takeuchi A Eto M Tatsugami K Shiota M Yamada H Kamiryo Y . Antitumor activity of recombinant Bacille Calmette-Guérin secreting interleukin-15-Ag85B fusion protein against bladder cancer. Int Immunopharmacol. (2016) 35:327–31. doi: 10.1016/j.intimp.2016.03.007, PMID: 27093372

[183] Bakula M Hudolin T Knezevic N Zimak Z Andelic J Juric I . Intravesical gemcitabine and docetaxel therapy for BCG-naïve patients: A promising approach to non-muscle invasive bladder cancer. Life (Basel). (2024) 14:789. doi: 10.3390/life14070789, PMID: 39063544 PMC11278229

